# Metal-free hypervalent iodine/TEMPO mediated oxidation of amines and mechanistic insight into the reaction pathways†

**DOI:** 10.1039/c8ra07451h

**Published:** 2018-09-14

**Authors:** Ajay H. Bansode, Gurunath Suryavanshi

**Affiliations:** Chemical Engineering & Process Development Division, CSIR-National Chemical Laboratory Dr Homi Bhabha Road Pune-411008 India gm.suryavanshi@ncl.res.in; Academy of Scientific and Innovative Research (AcSIR) New Delhi 110 025 India

## Abstract

A highly efficient metal free approach for the oxidation of primary and secondary amines to their corresponding aldehydes and ketones using PhI(OAc)_2_ in combination with a catalytic amount of TEMPO as an oxidizing agent is described. This protocol is rapid and provides diverse products under milder reaction conditions in excellent yields. In addition, the mechanistic study is well demonstrated by spectroscopic methods.

## Abstract 2

A highly efficient, metal free rapid protocol studied mechanistically the oxidation of primary and secondary amines to their corresponding carbonyl compounds using PhI(OAc)_2_ and catalytic TEMPO to provide diverse products in excellent yields.

## Graphical abstract

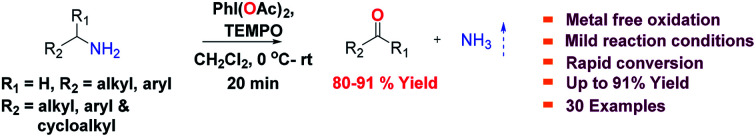

## Introduction

Oxidation reactions to access carbonyl functional groups are fundamental transformations and play a most significant role in synthetic organic chemistry. Carbonyl functionalities serve as versatile building blocks in functional group interconversions, and synthesis of complex molecules and are widely present in natural products and biologically active compounds. The conventional way to synthesize aldehydes and ketones involves oxidation of primary and secondary alcohols,^1^ which has been successfully exploited in academic and industrial research. In an alternative method, amine precursors were also successfully used to access carbonyl compounds due to their ability to undergo oxidation reactions, and their natural and commercial availability. The oxidation of amines is also used as a powerful tool to produce different synthetic intermediates: imines, nitriles, oximes and amides.^2^ In the last two decades, several protocols reported the synthesis of carbonyl compounds from amines using metal reagents/catalysts such as KMnO_4_,^3^ ZnCr_2_O_7_,^4^ nicotinium dichromate,^5^ palladium,^6^ copper^7^ and ruthenium.^8^ These traditional methods suffer from their own limitations, such as use of stoichiometric amounts of reagents/catalysts, inherent toxicity of metals, high temperature and limited substrate scope.

**Scheme 1:**
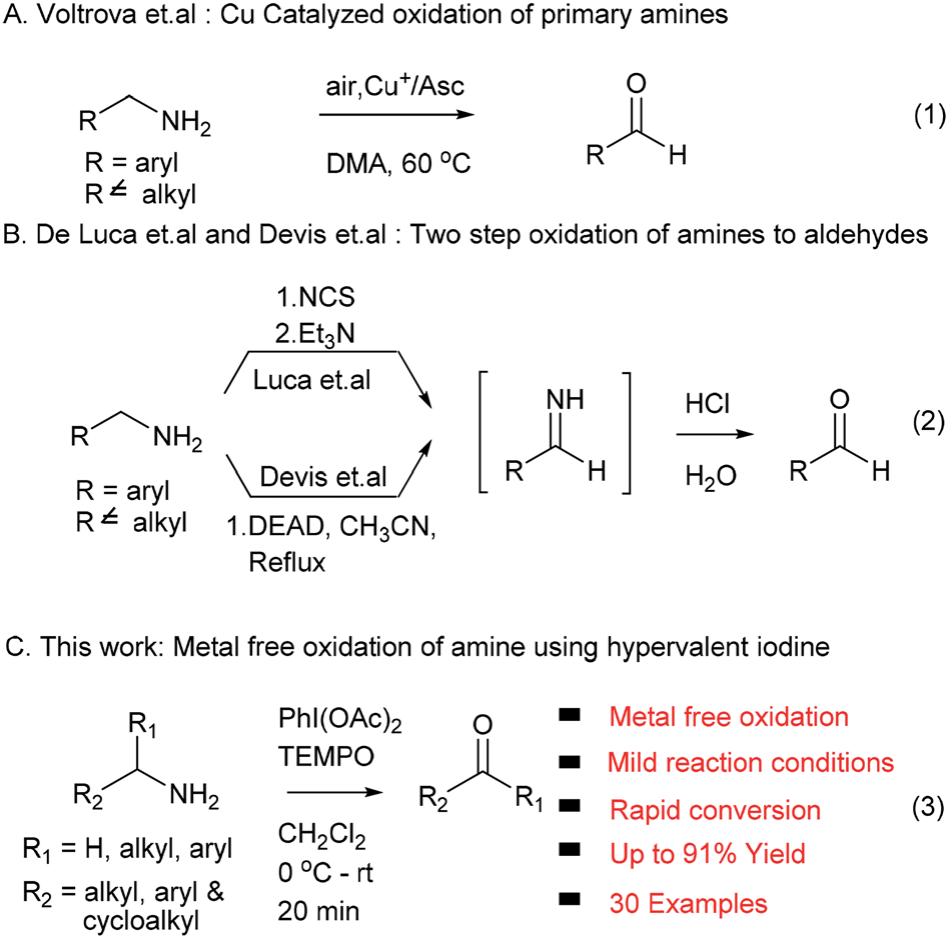
Oxidation of amine to aldehyde.

In addition, Voltrova *et al.* showed that catalytic copper and ascorbic acid in open air condition can be used for oxidation of amines, which is limited to primary amine substrates (eqn (1), Scheme 1).^9^ De Luca *et al.* and Davis *et al.* developed metal free methods using NCS/Et_3_N and DEAD *via* an imine intermediate in the classical way. These two methods required harsh reaction conditions and an extra hydrolysis step to access the target carbonyl compound (eqn (2), Scheme 1).^10,11^

In continuation of our interest in development of new methodologies using PhI(OAc)_2_ ^12^ and to address issues associated with existing protocols for the conversion of amines to carbonyl compounds, herein we report an efficient, rapid, mild, metal free and environment friendly protocol involving non-metallic, less toxic and affordable hypervalent iodine (PhI(OAc)_2_) in combination with TEMPO as an oxidizing agent for the first time without any external oxygen source (eqn (3), Scheme 1).^13,14^

Hypervalent iodine compounds such as (diacetoxyiodo)benzene (PhI(OAc)_2_), [bis(trifluoroacetoxy)] benzene (PhI(OCOCF_3_)_2_), Dess–Martin periodinane (DMP), 2-iodoxybenzoic acid (IBX) and their derivatives were extensively used as oxidants and co-oxidants in organic transformations.^15,16^ Using hypervalent iodine reagent oxidation of amine was well established for conversion to imines,^17*a*–*e*^ aldehydes,^18*a*,*b*^ ketones and aromatization of pyrrolidine, dihydropyridine rings.^19^ Very recently, Galletti *et al.* reported NaIO_4_/TEMPO mediated conversion of benzylamines to carbonyls *via* imine formation and *in situ* hydrolysis using H_2_O/CH_3_CN as solvent.^20^ However, these methods suffer from limitations such as higher temperature, long reaction time and sequence, substrate selectivity and extra step for imine hydrolysis to form an aldehyde.^17–20^ In this context we envisioned that, PhI(OAc)_2_ in combination with TEMPO used as an oxidizing agent to convert amine functional groups into corresponding carbonyl compounds at room temperature, with short reaction time and no use of external oxygen source (such as H_2_O or O_2_) with excellent yields.

## Result and discussion

To test our hypothesis, initially, *p*-methoxy benzyl amine (1a) was treated with PhI(OAc)_2_ (1 equiv.) and TEMPO (1 equiv.) in anhydrous CH_2_Cl_2_ for 30 min at 0 °C, which furnished the *p*-methoxy benzaldehyde (2a) in 40% yield along with 50% of unreacted amine 1a (entry 1), when we raised the reaction temperature to rt, the expected *p*-methoxy benzaldehyde (2a) was obtained in 65% yield along with decomposition of the starting material 1a in 30 min (entry 2). Hence, we carried out the same reaction for 10 min at 0 °C followed by room temperature for another 10 min, to our delight, this small modification to the reaction conditions afforded the desired oxidation product 2a in the excellent yield of 90% (entry 3). The output of the reaction was not much affected when we reduced the amount of TEMPO (0.5 equiv.), but the yield was dropped to 40% using 0.5 equiv. of PhI(OAc)_2_ (entries 4 and 5). Further reduction of TEMPO to 0.25 and 0.1 equiv. also furnished the product in good yields (entries 6 and 7). Then we verified the reaction without using TEMPO or PhI(OAc)_2,_ which led to the unchanged starting material (1a) (entries 8 and 9). Ultimately, the reaction conditions of PhI(OAc)_2_ (1 equiv.) and TEMPO (0.1 equiv.) in anhydrous CH_2_Cl_2_ for 10 min at 0 °C followed by room temperature for another 10 min were found to be ideal for this transformation (entry 7) (Table 1).

**Table 1:** Optimization of reaction conditions^a^

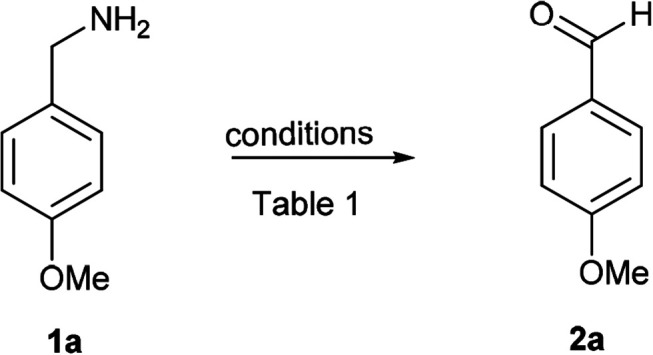				
Entry	PhI(OAc)_2_ (equiv.)	TEMPO (equiv.)	Temp (time)^b^	Yield^b^
1	1	1	0 °C (30 min)	40
2	1	1	rt (30 min)	65
3	1	1	0 °C to rt (20 min)	90
4	1	0.5	0 °C to rt (20 min)	90
5	0.5	1	0 °C to rt (20 min)	40
6	1	0.25	0 °C to rt (20 min)	90
**7**	**1**	**0.1**	**0 °C to rt (20 min)**	**90**
8	1	—	0 °C to rt (20 min)	NR^c^
9	—	1	0 °C to rt (20 min)	NR^c^

a Reaction conditions unless otherwise specified: 1a (1 equiv.), PhI(OAc)_2_ (1 equiv.), TEMPO (0.1 equiv.), 0 °C to rt, 20 min, anhydrous CH_2_Cl_2_.

b Isolated yields in percentage.

c Starting material (1a) recovered; rt = room temperature; NR: no reaction.

**Scheme 2:**
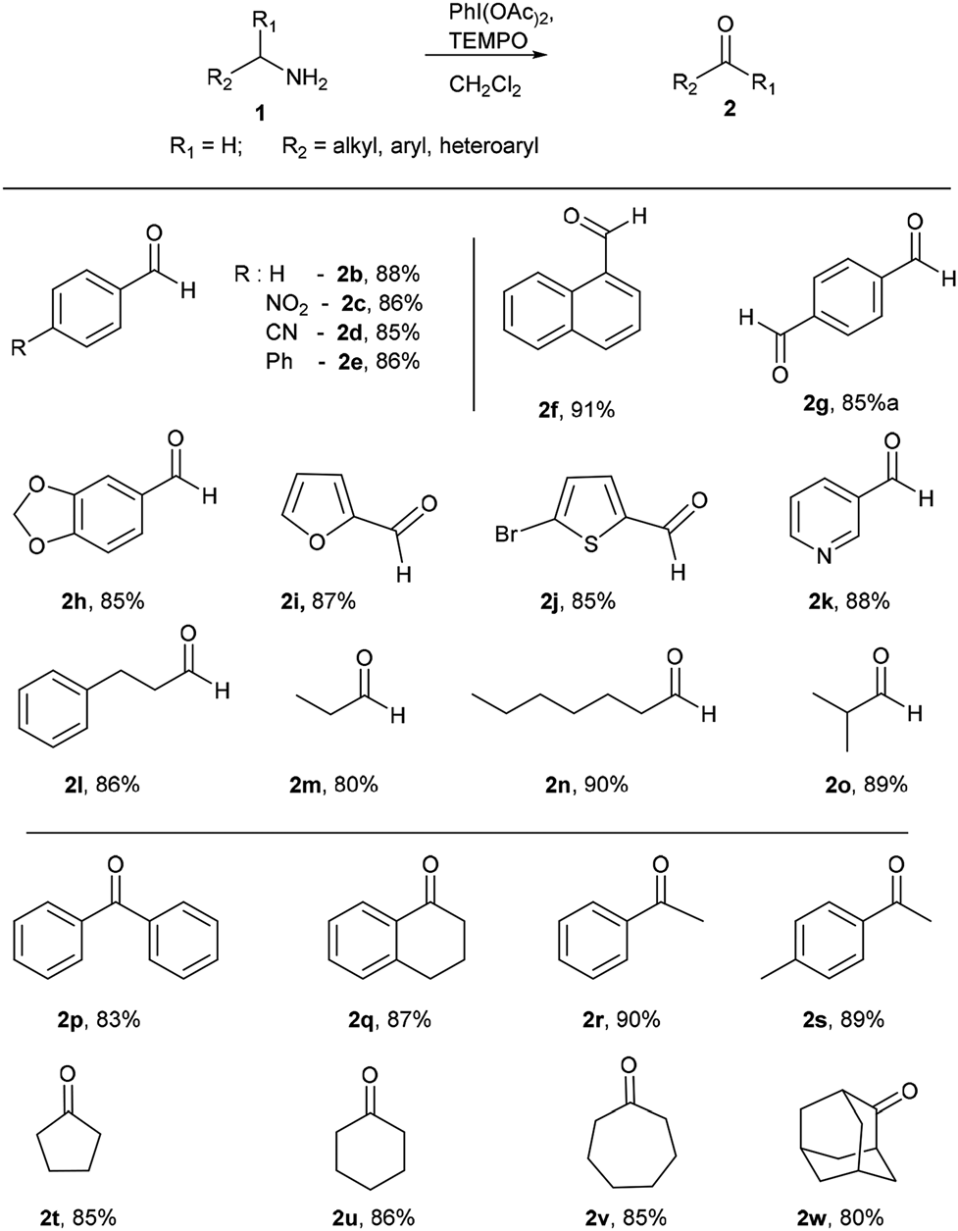
Oxidation of primary amines to aldehydes and ketones. Reaction conditions unless otherwise specified: amine 1 (1 equiv.), PhI(OAc)_2_ (1 equiv.), TEMPO (0.1 equiv.), 0 °C to rt, 20 min, anhydrous CH_2_Cl_2_, ^a^PhI(OAc)_2_ (2 equiv.), TEMPO (0.2 equiv.).

With optimized conditions in hand, initially, the substrate scope for this methodology was examined using a series of diverse aromatic and aliphatic primary amines (Scheme 2). Commercially available aryl and heteroaryl amines possessing electron donating and electron withdrawing substituents were provided corresponding aldehydes in excellent yields without significant discrimination in the output (2b–e). Naphthalen-1-ylmethanamine also well reacted and gave the corresponding aldehyde 2f in good yield of 91%. *p*-Xylylamine also a good substrate for this reaction, which delivered the terephthalaldehyde (2g) in 85% yield. The benzylamine with dioxolane protection is well tolerated in these reaction conditions and gave 2h in 85% yield. Heteroaryl amines like furan-2-ylmethanamine, (5-bromofuran-2-yl) methanamine and pyridin-3-yl-methanamine were well participated to give 2i, 2j and 2k respectively. To our delight, under identical conditions alkyl and dialkyl substituted amines (3-phenylpropan-1-amine, *n*-propylamine, *n*-heptylamines and isobutylamine) well tolerated and furnished corresponding aldehydes in excellent yields (2l–o). Next the reactivity of diverse alpha-disubstituted primary amine derivatives were examined to obtained corresponding ketones. Highly hindered alpha substituted benzyl amine and tetrahydronaphthalene derived amines provided corresponding ketones 2p and 2q in good yields of 83% and 87%. Phenyl and tolyl substituted amines gave 2r and 2s respectively. Cycloalkyl derived amines furnished 2t–v in good yields, even hindered 2-adamantyl amine was converted into 2-adamantanone (2w) in 80% yield.

**Scheme 3:**
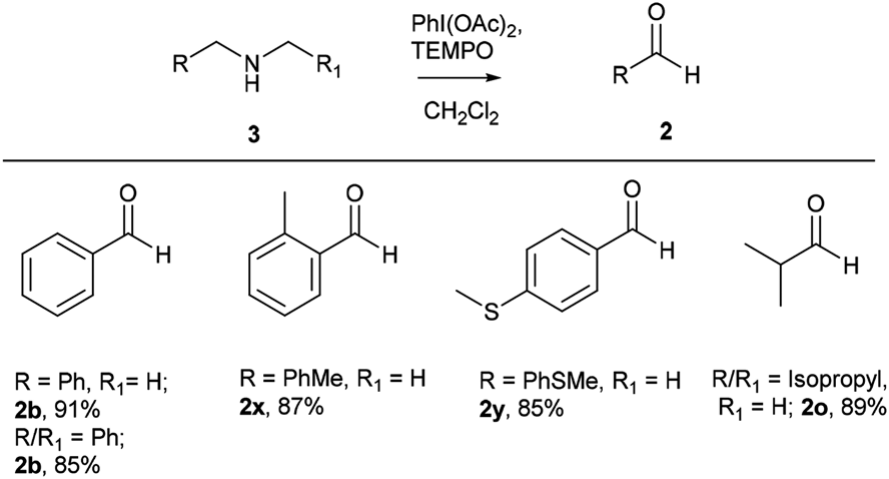
Oxidation of secondary amines to aldehydes. Reaction conditions unless otherwise specified: amine 1 (1 equiv.), PhI(OAc)_2_ (2 equiv), TEMPO (0.2 equiv), anhydrous CH_2_Cl_2_, 0 °C to rt, 20 min.

**Scheme 4:**
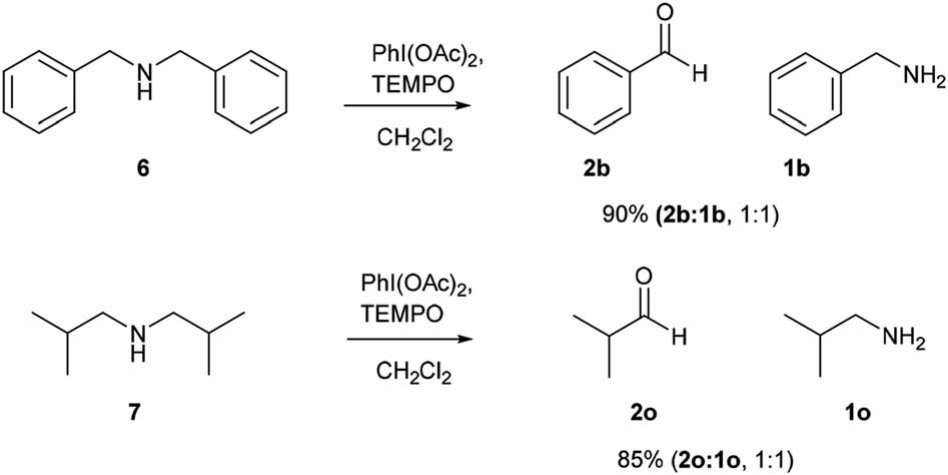
Comparative study on symmetrical secondary amines. Reaction conditions unless otherwise specified: amine 1 (1 equiv.), PhI(OAc)_2_ (1 equiv.), TEMPO (0.1 equiv.), anhydrous CH_2_Cl_2_, 0 °C to rt, 20 min.

After successful studies involving primary amines, we were curious to know the reactivity of secondary amines in this oxidation protocol. Several alkyl–aryl and dialkyl amines were subjected to slightly modified reaction conditions of 2 equiv. of PhI(OAc)_2_ and 0.2 equiv. of TEMPO in dry CH_2_Cl_2_. The oxidations of *N*-methyl-aryl amine substrates were quite interesting, which would generate possible two products of aryl-aldehydes and formaldehyde based on the choice of oxidation sites. To our surprise, aryl-aldehydes were obtained exclusively in good yield (2b, 2x and 2y), which could be due to the more reactivity of the benzylic position of substrates. This selectivity is in contrast with other oxidizing agents used in earlier reports.^3–11^ Isobutyl-methylamine also well reacted under identical conditions to provide corresponding isobutyraldehyde 2o in excellent yield of 89% (Scheme 3). Oxidation of symmetrical amines such as dibenzylamine using PhI(OAc)_2_ (1 equiv) and TEMPO (0.1 equiv) in dry CH_2_Cl_2_ provided the mixture of benzaldehyde (2b) and benzyl amine (1b) in 1 : 1 ratio in the good yield of 90% yield. Equivalent results were obtained in the oxidation of diisobutylamine (7) and gave isobutyraldehyde (2o) and isobutylamine (1o) in 1 : 1 ratio in excellent yield of 85% (Scheme 4).

Based on earlier reports^21^ and results obtained in this work, a plausible reaction mechanism was proposed. Initially, TEMPO was converted into oxoammonium species A using PhI(OAc)_2_ as oxidant. The reaction of benzylamine (1) with oxoammonium species A could provide the intermediate B*via* attack of lone pair of nitrogen from benzyl amine on electron deficient nitrogen of oxoammonium A. Then, intermediate B could undergo reductive elimination which provide the reactive imine C through the abstraction of proton from benzylic position of intermediate B. Further PhI(OAc)_2_ was used for oxidation of hydroxyamine species I to regenerate oxoammonium species A and re-enters in catalytic cycle, expels iodobenzene and acetic acid which was used in further reaction sequence. Imine C then converted into corresponding aldehyde *via* two probable pathways. In the path-I, imine intermediate C will react with acetic acid and forms the amino acetate intermediate D. Intermediate D would react with another 1 mole of acetic acid to give the corresponding acetate intermediate E, which further reacts to give desired aldehyde 2*via* releasing the acetic anhydride and ammonia. In an alternate path II, imine C will react with benzylamine (1b) with exertion of ammonia to provide secondary imine F,^22^ which will react with acetic acid to provide the amino-acetate intermediate G. Under acidic condition as in the path I, intermediate G delivers the aldehyde 2 and benzylamine which re-enters the catalytic cycle.

**Scheme 5:**
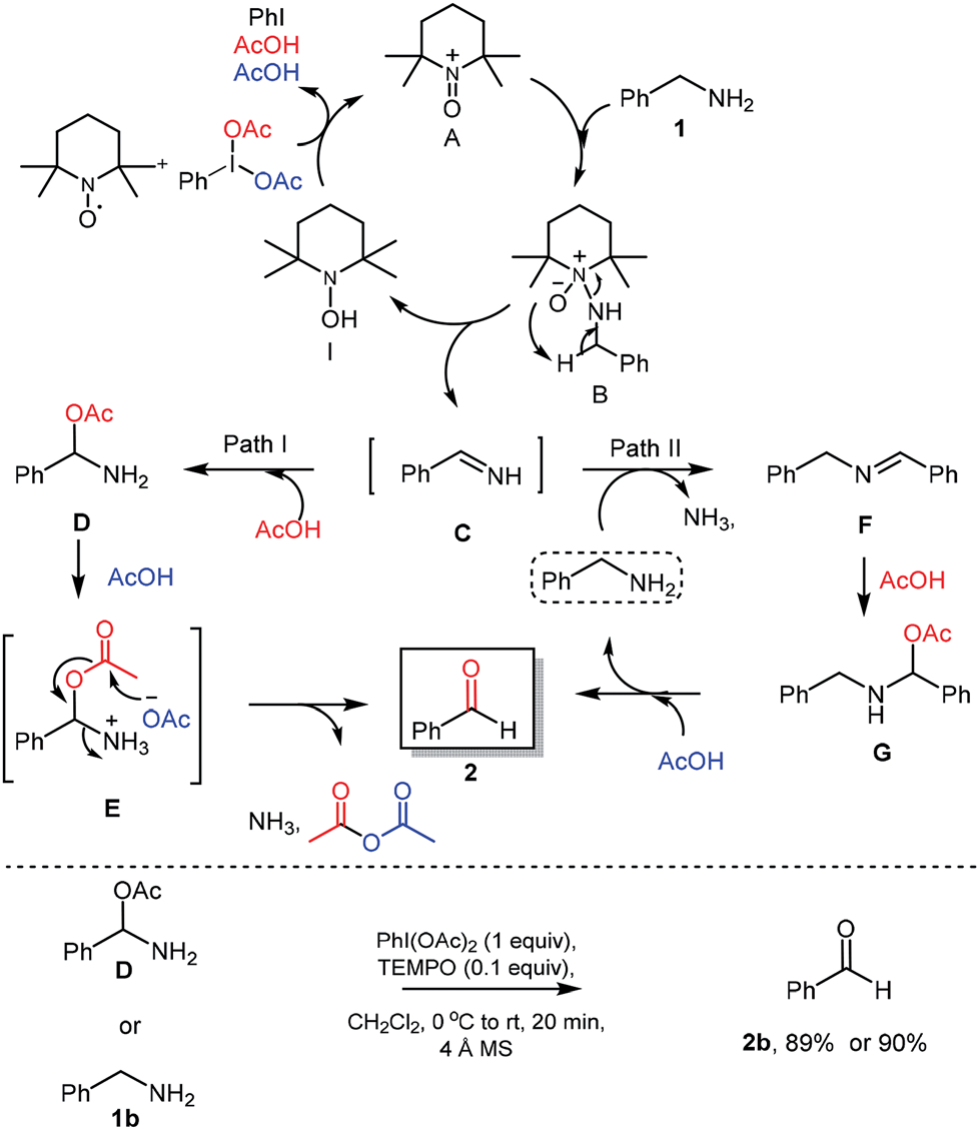
Plausible reaction mechanism & control experiments.

To further understand the proposed mechanistic sequence, a few supporting experiments were carried out and described in Scheme 5. To verify, the probable formation of amino acetate intermediate D, we have prepared the same using known literature procedure^23^ and subjected to standard reaction conditions of PhI(OAc)_2_ (1 equiv.) and TEMPO (0.1 equiv.). To our delight, the expected aldehyde 2 was isolated in 90% yield. The possible hydrolysis of imine intermediate C, which could directly deliver the aldehyde without the intervention of path I and/or path II was established by performing the reaction of amino acetate or amine under optimized conditions using 4 Å-MS.

The formation of imine C and amino acetate D intermediates and release of acetic anhydride and ammonia as by-products in this reaction was established by time dependent ^1^H NMR analysis. A standard reaction using benzyl amine in a sealed NMR tube in CDCl_3_ under inert atmosphere was studied.

**Fig. 1:**
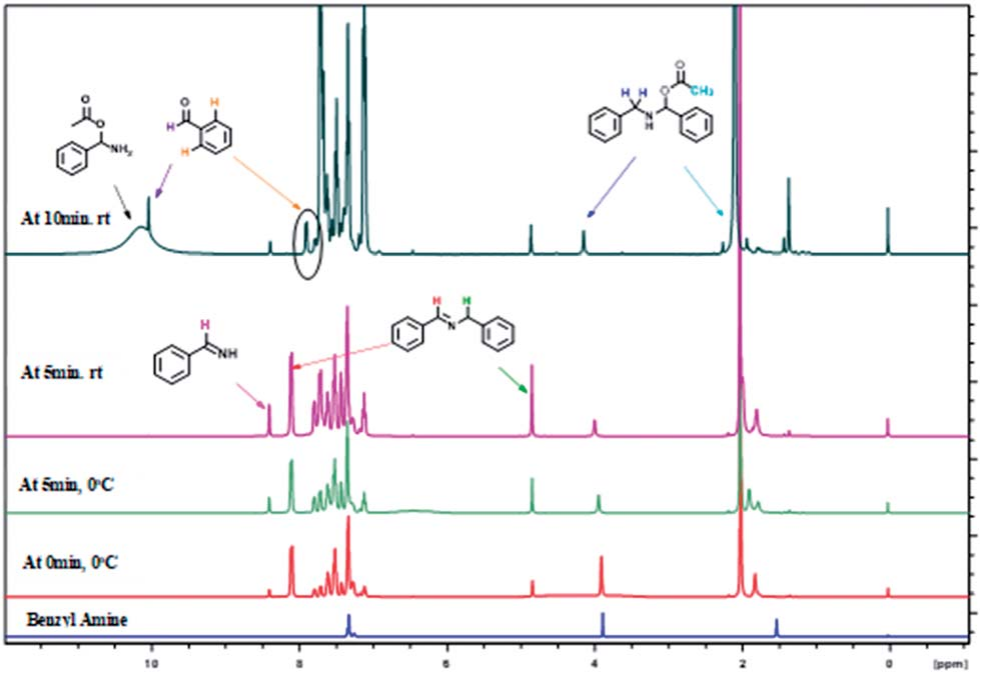
Time dependent ^1^H NMR experiment for mechanistic determination.

Analysis of ^1^H NMR at 0 h without adding reagents were analysed, showed peaks at 1.5 (s, –NH_2_), 3.97(s, benzylic –CH_2_), 7.2–7.6 (m, aromatic 5H), belongs to benzyl amine. To the same NMR tube, PhI(OAc)_2_ (1 equiv.) and TEMPO (0.1 equiv.) were added at 0 °C, as soon as the addition was completed, ^1^H NMR was recorded, in which we found that, peak at 3.97 ppm belongs to benzylic proton of benzyl amine was disappeared and new peaks for intermediates were clearly indicative. We found out a singlet at 10.1 ppm, which clearly show that the peak of benzaldehyde proton and doublet at 7.98 ppm is for *ortho* proton of benzaldehyde. In addition, we found signals related to imine intermediates, amino acetate intermediate as well while performing the analysis (as shown in Fig. 1).

In addition, we carried out GC-MS study to prove our hypothesis that oxygen is not coming from out sources and it is from PhI(OAc)_2_. We performed reaction in air tight GC-MS vial to avoid the contamination with atmospheric oxygen. To our delight, we found out peak corresponding to benzaldehyde and intermediate F. Generation of benzaldehyde in reaction mixture itself, without additional hydrolysis clearly indicates that no external oxygen source is required and PhI(OAc)_2_ is acting as an oxygen source in this reaction. In addition, release of ammonia was detected using well-known ammonia test.

**Fig. 2:**
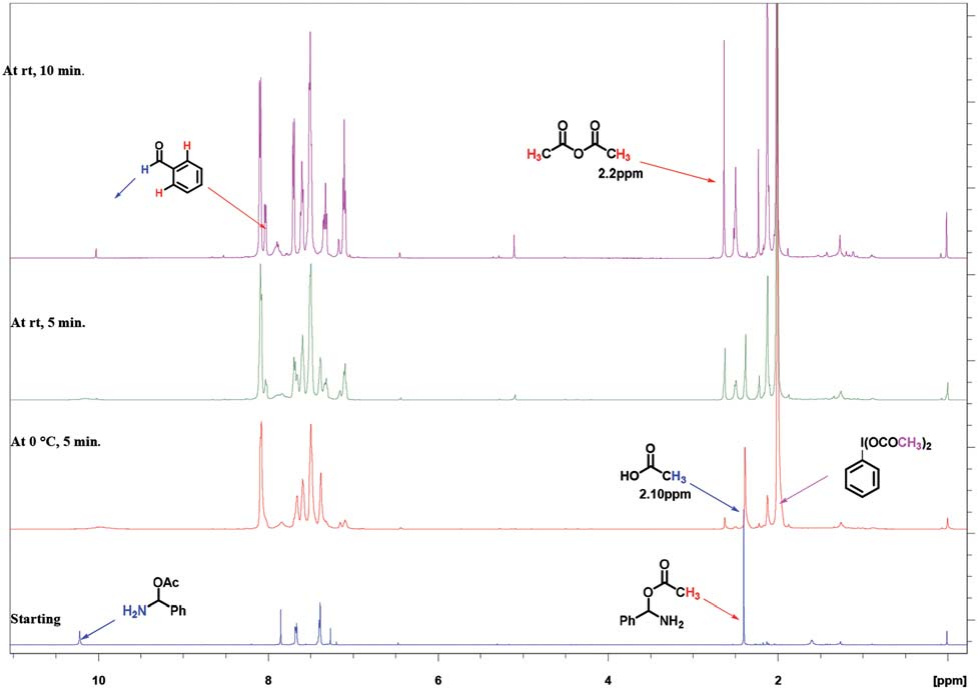
Time dependent ^1^H NMR experiment for mechanistic determination on intermediate.

Initially, we took ^1^H NMR of our intermediate amino acetate D in CDCl_3_, which showed peaks at 2.4 ppm (s, –CH_3_), 7.68 ppm (s, benzylic –CH), 10.22 ppm (bs, NH_2_). In same NMR tube we added PhI(OAc)_2_ (1 equiv.) and TEMPO (0.1 equiv.) at 0 °C, as soon as addition completed we took ^1^H NMR. We found out that peak at 2.4 (s, –CH_3_), 7.68 (s, benzylic –CH), 10.22 (bs, NH_2_) was disappeared and new peaks at 2.10 ppm for acetic acid, 2.2 ppm for acetic anhydride are clearly indicative (Fig. 2).

**Fig. 3:**
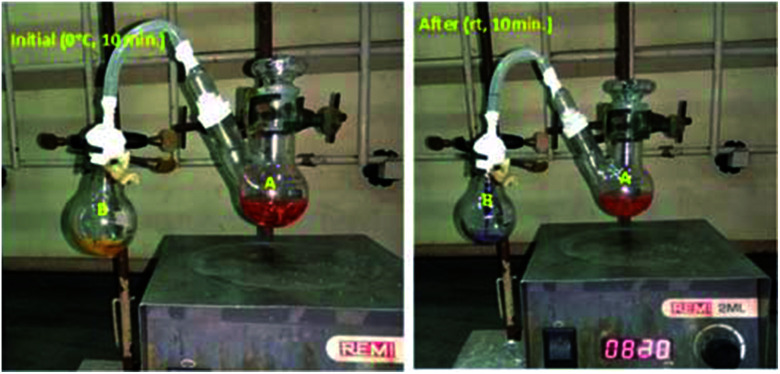
Mechanistic study (ammonia trapping experiments).

Our main goal is to find out in which form –NH_2_ is going out either ammonia or amide. To prove our side product, we carried out ammonia trapping experiment. In this we took two neck RB (A) equipped with magnetic needle contenting Benzyl amine (500 mg, 4.67 mmol) and dry CH_2_Cl_2_ (5 mL). Other side single neck RB (B) having water and small pieces of pH paper. To connect this two RB's, we used silicon pipe having one side connecter connected to two neck RB (A) and other side dropper which dipped in single neck RB (B) (as shown in picture below). Reaction start with addition of PhI(OAc)_2_ (1.5 g, 4.67 mmol) and TEMPO (72 mg, 0.467 mmol) at 0 °C for 10 minutes followed by 10 minutes at room temperature. When we did reaction at 0 °C for 10 minutes neither bubbling nor color change observed in RB-B. When we increase temperature form 0 °C to room temperature of RB-A and within 5 minutes in RB-B bubbling as well as color change of pH paper from yellow to purple was observed. With time span of 5 to 10 minutes at room temperature vigorous bubbling and color changed to purple. In addition to this intense smell of ammonia was observed. Bubbling and color change of pH paper yellow to purple clearly indicate formation of ammonia (pH: 11.6 and purple on pH paper) (Fig. 3).

## Experimental section

### General procedure

All reaction carried out under nitrogen atmosphere with dry solvents kept anhydrous condition. Anhydrous CH_2_Cl_2_ was distilled from P_2_O_5_. Yields are calculated by chromatographical isolation. Thin layer chromatography was performed using commercially prepared silica gel plates, and visualization was effect with short wavelength UV light (254 nm). Column chromatography performed on 100–200 mesh size silica gel.

The ^1^H and ^13^C NMR spectra were recorded on Bruker advance 500 (^1^H 500 MHz, ^13^C 125 MHz) or Bruker advance 400 (^1^H 400 MHz, ^13^C 100 MHz) or Bruker advance 200(^1^H 200 MHz, ^13^C 50 MHz), otherwise mentioned. Deuterated solvent CDCl_3_ + CCl_4_ (70 : 30) were used as internal standard and singlet at 96.1 ppm in ^13^C NMR corresponds to carbon of CCl_4_. Solvent signal was used as reference for ^13^C NMR (CDCl_3_, 77.0 ppm) or ^13^C NMR (DMSO-d_6_, 40.0 ppm). The following abbreviations were used to explain the multiplicities: s = singlet, d = doublet, t = triplet, q = quartet, m = multiple, br = broad. Chemical shifts are reported in ppm and referenced to residual solvent peak or TMS. Coupling constants are reported in hertz. High resolution mass spectra were recorded on a double focusing magnetic sector mass spectrometer using EI at 70 eV. The GC analysis were done using Agilent 7890A GC coupled with mass detector. All reagents were used as obtained commercially. The PhI(OAc)_2_ and TEMPO were purchased by Sigma Aldrich.

#### General procedures to convert primary amine to aldehyde

To the solution of amine (1.0 mmol) was added PhI(OAc)_2_ (1 mmol) in 2 mL of dry CH_2_Cl_2_ at 0 °C under nitrogen atmosphere and followed by addition of TEMPO (0.1 mmol) at 0 °C. The resulting mixture was stirred at 0 °C for 10 min. Then reaction mixture was gradually allowed to stir at room temperature for 10 min. The progress of reaction was monitored by TLC. The reaction mixture was quenched by water (5 mL) and extracted with CH_2_Cl_2_ (3 × 5 mL). The organic layers were combined, wash with brine, dried over Na_2_SO_4_ and concentrated under reduced pressure. The residue was purified by column chromatography on silica gel and 2% ethyl acetate in petroleum ether as an eluting solvent to afford the desired aldehyde.

#### General procedures to convert secondary amine to aldehyde and amine

To the solution of amine (1.0 mmol) was added PhI(OAc)_2_ (1 mmol) in 2 mL of dry CH_2_Cl_2_ at 0 °C under nitrogen atmosphere and followed by addition of TEMPO (0.1 mmol) at 0 °C. The resulting mixture was stirred at 0 °C for 10 min. Then reaction mixture was gradually allowed to stir at room temperature for 10 min. The progress of reaction was monitored by TLC. The reaction mixture was quenched by water (5 mL) and extracted with CH_2_Cl_2_ (3 × 5 mL). The organic layers were combined, wash with brine, dried over Na_2_SO_4_ and concentrated under reduced pressure. The residue was purified by column chromatography on silica gel and 2% ethyl acetate in petroleum ether as an eluting solvent to afford the desired aldehyde and 25% ethyl acetate in petroleum ether as an eluting solvent to afford the desired amine.

#### General procedures to convert secondary amine to aldehyde

To the solution of amine (1.0 mmol) was added PhI(OAc)_2_ (2 mmol) in 4 mL of dry CH_2_Cl_2_ at 0 °C under nitrogen atmosphere and followed by addition of TEMPO (0.2 mmol) at 0 °C. The resulting mixture was stirred at 0 °C for 10 min. Then reaction mixture was gradually allowed to stir at room temperature for 10 min. The progress of reaction was monitored by TLC. The reaction mixture was quenched by water (5 mL) and extracted with CH_2_Cl_2_ (3 × 5 mL). The organic layers were combined, wash with brine, dried over Na_2_SO_4_ and concentrated under reduced pressure. The residue was purified by column chromatography on silica gel and 2% ethyl acetate in petroleum ether as an eluting solvent to afford the desired aldehyde.

##### *p*-Anisaldehyde (2a)^24^

54 mg (90%); colourless oil; ^1^H NMR (200 MHz, CDCl_3_, ppm): *δ* 3.89 (s, 3H), 6.99 (m, *J* = 8.84 Hz, 2H), 7.83 (m, *J* = 8.84 Hz, 2H), 9.88 (s, 1H); ^13^C NMR (50 MHz, CDCl_3_, ppm): *δ* 55.47, 114.28, 130.06, 131.93, 164.54, 190.39; HRMS (ESI) calcd for C_8_H_8_O_2_ [M + H]^+^ 137.0597; found 137.0599.

##### Benzaldehyde (2b)^24^

194 mg, 88%; colourless oil; ^1^H NMR (200 MHz, CDCl_3_, ppm): *δ* 7.46–7.68 (m, 4H), 7.78–7.98 (m, 2H), 10.02 (s, 1H); ^13^C NMR (50 MHz, CDCl_3,_ ppm): *δ* 128.9, 129.7, 134.3, 136.5, 191.9; HRMS (ESI) calcd for C_7_H_6_O [M + H]^+^ 107.0491; found 107.0495.

##### 4-Nitrobenzaldehyde (2c)^28^

20 mg, 86%; white solid; ^1^H NMR (200 MHz, CDCl_3_, ppm): *δ* 8.09 (m, *J* = 8.84 Hz, 2H), 8.41 (m, *J* = 8.72 Hz, 2H), 10.17 (s, 1H); ^13^C NMR (50 MHz, CDCl_3_, ppm): *δ* 124.3, 130.5, 140.1, 151.2, 190.3; HRMS (ESI) calcd for C_7_H_5_NO_3_ [M + H]^+^ 152.0342; found 152.0703.

##### 4-Formylbenzonitrile (2d)^26^

50 mg, 85%; white solid; ^1^H NMR (200 MHz, CDCl_3_, ppm): *δ* 7.83–7.92 (m, 2H), 7.97–8.05 (m, 2H), 10.11 (s, 1H); ^13^C NMR (50 MHz, CDCl_3_, ppm) *δ* 117.6, 117.7, 129.9, 132.9, 138.8, 190.7; HRMS (ESI) calcd for C_8_H_5_NO [M + H]^+^ 132.0444; found 132.0443.

##### 4-Phenylbenzaldehyde (2e)^27^

85 mg, 86%; white solid; ^1^H NMR (200 MHz, CDCl_3_, ppm): *δ* 7.38–7.52 (m, 3H), 7.58–7.67 (m, 2H), 7.69–7.78 (m, 2H), 7.89–7.98 (m, 2H), 10.04 (s, 1H); ^13^C NMR (50 MHz, CDCl_3_): *δ* 127.4, 127.7, 128.5, 129.1, 130.3, 135.3, 139.8, 147.2, 191.93; HRMS (ESI) calcd for C_13_H_10_O [M + H]^+^ 183.0804; found 183.0804.

##### 1-Naphthaldehyde (2f)^24^

195 mg, 91%; colourless oil; ^1^H NMR (200 MHz, DMSO-d_6_, ppm): *δ* 7.54–7.84 (m, 3H), 7.96–8.39 (m, 3H), 9.18 (d, *J* = 8.34 Hz, 1H), 10.42 (s, 1H); ^13^C NMR (101 MHz, DMSO-d_6_, ppm): *δ* 124.6, 125.9, 127.4, 129.2, 129.5, 130.2, 131.3, 133.8, 135.7, 137.2, 194.9; HRMS (ESI) calcd for C_11_H_8_O [M + H]^+^ 157.0648; found 157.0647.

##### Terephthalaldehyde (2g)^32^

100 mg, 85%; white solid; ^1^H NMR (200 MHz, CDCl_3_, ppm): *δ* 8.06 (s, 4H), 10.14 (s, 2H); ^13^C NMR (50 MHz, CDCl_3_, ppm): *δ* 130.1, 140.0, 191.1; HRMS (ESI) calcd for C_8_H_6_O_2_ [M + H]^+^ 135.0441; found 135.0439.

##### Piperonal (2h)^29^

45 mg, 85%; white solid; ^1^H NMR (500 MHz, CDCl_3_, ppm): *δ* 6.03–6.11 (m, 2H), 6.88–6.96 (m, 1H), 7.29–7.35 (m, 1H) 7.36–7.44 (m, 1H), 9.77–9.83 (m, 1H); ^13^C NMR (126 MHz, CDCl_3_, ppm): *δ* 102.0, 106.9, 108.3, 128.49, 131.9, 148.7, 153.0, 189.9; HRMS (ESI) calcd for C_8_H_6_O_3_ [M + H]^+^ 151.0390; found 151.0390.

##### Furfural (2i)^24^

43 mg, 87%; colourless oil; ^1^H NMR (200 MHz, CDCl_3_, ppm): *δ* 6.62 (dd, *J* = 3.54, 1.64 Hz, 1H) 7.26 (d, *J* = 3.54 Hz, 1H), 7.61–7.79 (m, 1H), 9.67 (s, 1H); ^13^C NMR (50 MHz, CDCl_3_, ppm): *δ* 112.5, 120.7, 147.9, 153, 177.6; GC MS (ESI) calcd for C_5_H_4_O_2_ [M]+ 97; found 97.

##### 5-Bromo-2-thiophenecarboxaldehyde (2j)

15 mg, 85%; colourless oil; ^1^H NMR (200 MHz, CDCl_3_, ppm) *δ* 7.18–7.23 (m, 1H), 7.53–7.57 (m, 2H), 9.78–9.80 (m, 1H); ^13^C NMR (50 MHz, CDCl_3_, ppm): *δ* 125.0, 131.5, 136.7, 145.2, 181.81; HRMS (ESI) calcd for C_5_H_3_BrOS [M + H]^+^ 190.9161; found 190.9162.

##### 3-Pyridinecarboxaldehyde (2k)^25^

200 mg, 88%; colourless oil; ^1^H NMR (200 MHz, CDCl_3_, ppm): *δ* 7.51 (dd, *J* = 7.71, 4.93 Hz, 1H), 8.20 (d, *J* = 7.83 Hz, 1H), 8.87 (dd, *J* = 4.61, 1.07 Hz, 1H), 9.03–9.15 (m, 1H), 10.14 (s, 1H); ^13^C NMR (50 MHz, CDCl, ppm) *δ* 124.1, 131.4, 135.8, 152.1, 154.7, 190.8; HRMS (ESI) calcd for C_6_H_5_NO [M + H]^+^ 108.0444; found 108.0446.

##### 3-Phenyl propan-1-al (2l)^26^

40 mg, 86%; colourless oil; ^1^H NMR (200 MHz, CDCl_3,_ ppm): *δ* 2.70–2.81 (m, 2H), 2.89–2.99 (m, 2H), 7.14–7.25 (m, 5H), 9.79 (t, *J* = 1.26 Hz, 1H); ^13^C NMR (50 MHz, CDCl_3_, ppm): *δ* 28.2, 45.3, 126.3, 128.3, 128.4, 128.5, 128.6, 140.3, 201.1; HRMS (ESI) calcd for C_9_H_10_O [M + Na]^+^ 157.0624; found 157.0648.

##### Propionaldehyde (2m)

100 mg, 80% {isolated by distillation}; colourless oil; ^1^H NMR (200 MHz, CDCl_3_, ppm) *δ* 1.11 (t, *J* = 7.39 Hz, 3H), 2.39–2.56 (m, 2H), 9.80 (s, 1H); ^13^C NMR (50 MHz, CDCl_3,_ ppm): *δ* 6, 37.2, 203; GC-MS (ESI) calcd for C_3_H_6_O [M] 58.04; found 58.0.

##### Heptaldehyde (2n)^23^

143 mg, 90%; colourless oil; ^1^H NMR (500 MHz, CDCl_3_, ppm): *δ* 0.90 (t, *J* = 6.48 Hz, 3H), 1.29–1.37 (m, 6H), 1.57–1.69 (m, 2H), 2.42 (td, *J* = 7.25, 1.53 Hz, 2H), 9.77 (s, 1H); ^13^C NMR (126 MHz, CDCl_3_, ppm): *δ* 14, 22.1, 22.4, 28.8, 31.5, 43.9, 202.3; HRMS (ESI) calcd for C_7_H_14_O [M + H]^+^ 115.1117; found 115.0868.

##### Isobutyraldehyde (2o)^27^

150 mg, 89%; colourless oil; ^1^H NMR (500 MHz, CDCl_3_, ppm): *δ* 1.09–1.11 (m, 6H), 2.40 (dtd, *J* = 14.11, 7.06, 7.06, 1.14 Hz, 1H), 9.57–9.80 (m, 1H); ^13^C NMR (126 MHz, CDCl_3_, ppm): *δ* 15.4, 40.9, 204.5; GC-MS (ESI) calcd for C_4_H_8_O [M] 73.0; found 73.0.

##### Benzophenone (2p)^23^

150 mg, 83%; white solid; ^1^H NMR (200 MHz, CDCl_3_, ppm): *δ* ppm 7.41–7.60 (m, 6H), 7.75–7.84 (m, 4H); ^13^C NMR (50 MHz, CDCl_3_, ppm): *δ* 128.2, 130.1, 132.3, 137.7, 196.4; HRMS (ESI) calcd for C_13_H_11_O [M + H]^+^ 183.0804; found 183.0803.

##### Tetralone (2q)^30^

75 mg, 87%; colourless oil; ^1^H NMR (200 MHz, CDCl_3_,ppm): *δ* 2.04–2.25 (m, 2H), 2.56–2.72 (m, 2H), 2.97 (t, *J* = 6.06 Hz, 2H), 7.16–7.35 (m, 2H), 7.38–7.51 (m, 1H), 8.02 (dd, *J* = 7.77, 1.20 Hz, 1H); ^13^C NMR (50 MHz, CDCl_3_, ppm): *δ* 23.32, 29.77, 39.13, 126.65, 127.28, 128.68, 132.68, 133.30, 144.30, 197.88; HRMS (ESI) calcd for C_10_H_10_O [M + H]^+^ 147.0804; found 147.0806.

##### Acetophenone (2r)^24^

112 mg, 90%; colourless oil; ^1^H NMR (200 MHz, CDCl_3_, ppm): *δ* 2.60 (s, 3H) 7.37–7.58 (m, 3H), 7.89–8.00 (m, 2H); ^13^C NMR (50 MHz, CDCl_3_, ppm): *δ* 26.50, 128.30, 128.53, 133.00, 137.19, 197.60; HRMS (ESI) calcd for C_8_H_8_O [M + H]^+^ 121.0648; found 113.0651.

##### 4-Methylacetophenone (2s)^28^

59 mg, 89%; colourless oil; ^1^H NMR (400 MHz, CDCl_3_, ppm): *δ* 2.40 (s, 3H), 2.55 (s, 3H), 7.23 (m, *J* = 7.93 Hz, 2H), 7.83 (m, *J* = 7.93 Hz, 2H); ^13^C NMR (101 MHz, DMSO-d_6_, ppm): *δ* 21.6, 26.4, 128.4, 129.2, 134.8, 143.6, 197.3; HRMS (ESI) calcd for C_9_H_10_O [M + H]^+^ 135.0804; found 135.0807.

##### Cyclopentanone (2t)^30^

100 mg, 85%; colourless oil; ^1^H NMR (400 MHz, CDCl_3_, ppm) *δ* 1.84–2.02 (m, 4H), 2.12 (t, *J* = 7.32 Hz, 4H); ^13^C NMR (101 MHz, CDCl_3_, ppm) *δ* 23.2, 38.3, 220.7; GC-MS (ESI) calcd for C_5_H_8_O [M] 85.0; found 85.0.

##### Cyclohexanone (2u)^26^

200 mg, 86%; colourless oil; ^1^H NMR (200 MHz, CDCl_3_, ppm) *δ* 1.67–1.79 (m, 2H), 1.80–1.97 (m, 4H), 2.27–2.39 (m, 4H); ^13^C NMR (50 MHz, CDCl_3_, ppm) *δ* 25.1, 27.0, 41.91, 211.39; GC-MS (ESI) calcd for C_6_H_10_O [M] 98.0; found 98.0.

##### Cycloheptanone (2v)^30^

45 mg, 85%; colourless oil; ^1^H NMR (200 MHz, CDCl_3_, ppm): *δ* 1.56–1.87 (m, 8H), 2.38–2.63 (m, 4H); ^13^C NMR (50 MHz, CDCl_3_, ppm): *δ* 24.36, 30.46, 43.80, 214.64; HRMS (ESI) calcd for C_7_H_12_O [M + H]^+^ 113.0961; found 113.0965.

##### 2-Adamantanone (2w)^31^

90 mg, 80%; white solid; ^1^H NMR (400 MHz, CDCl_3_, ppm): *δ* 1.9 (br. s, 2H), 2.0 (m, 5H), 2.1 (m, 5H), 2.6 (br. s, 2H); ^13^C NMR (50 MHz, CDCl_3_, ppm): *δ* 27.5, 36.3, 39.3, 47.0, 218.5; HRMS (ESI) calcd for C_10_H_14_O [M + H]^+^ 173.0937; found173.0123.

##### *o*-Tolualdehyde (2x)^28^

50 mg, 87%; colourless oil; ^1^H NMR (200 MHz, CDCl_3_, ppm): *δ* 2.70 (s, 3H), 7.23–7.56 (m, 3H), 7.83 (d, *J* = 7.33 Hz, 1H), 10.30 (s, 1H); ^13^C NMR (50 MHz, CDCl_3_, ppm): *δ* 19.6, 126.3, 131.8, 132.1, 133.7, 134.2, 140.6, 192.9; HRMS (ESI) calcd for C_8_H_8_O [M + H]^+^ 121.0648; found 121.648.

##### 4-(Methylthio)benzaldehyde (2y)^29^

30 mg, 85%; colourless oil; ^1^H NMR (200 MHz, CDCl_3_, ppm): *δ* 2.50 (s, 3H), 7.26–7.32 (m, 2H), 7.71–7.78 (m, 2H), 9.89 (s, 1H); ^13^C NMR (50 MHz, CDCl_3_, ppm): *δ* 14.7, 125.2, 130, 133, 147.9, 191.2; HRMS (ESI) calcd for C_8_H_8_OS [M + H]^+^ 153.0369; found 153.367.
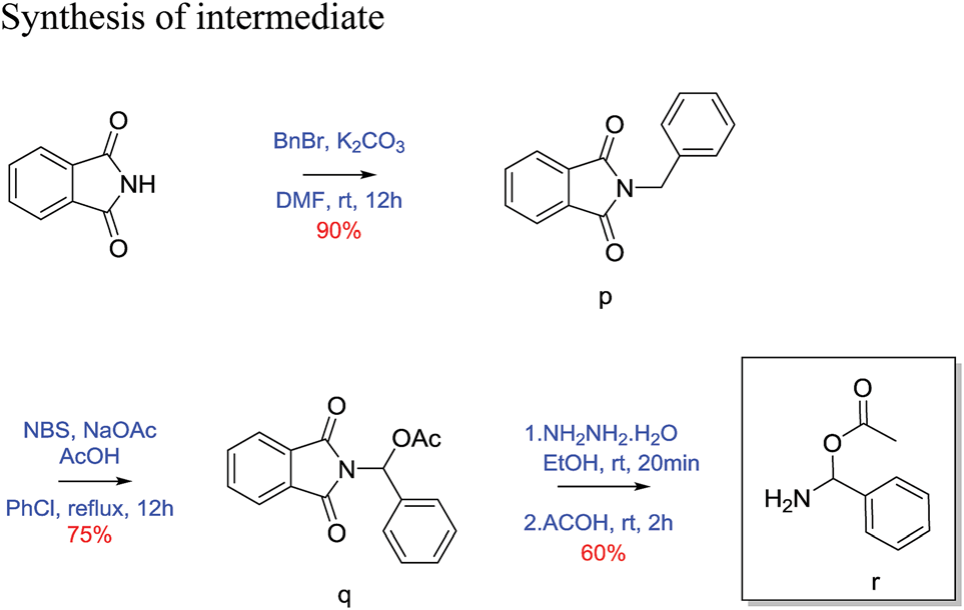


##### 2-Benzylisoindoline-1,3-dione (p)^23^

Phthalimide (1 g, 6.79 mmol) was taken in round bottom flask equipped with magnetic stirrer and dissolved in dry DMF (25 mL). K_2_CO_3_(1.87 g, 13.59 mmol) and benzyl bromide (1.162 g, 6.79 mmol) was added to above reaction mixture. Then the reaction mixture was stirred for 12 h at room temperature. Reaction was quenched with H_2_O (12 mL) and extracted with ethyl acetate (3 × 15 mL). The combined organic layer wash with brine and dried over Na_2_SO_4_. The crude product was then purified by column chromatography using silica (100–200 mesh) and 20% ethyl acetate/pet. ether as eluent gave 90% yield of benzyl protected phthalimide as white solid. 30 mg; ^1^H NMR (200 MHz, CDCl_3,_ ppm): *δ* 4.82 (s, 2H), 7.35 (m, 5H), 7.67 (m, 2H), 7.82 (m, 2H); ^13^C NMR (50 MHz, CDCl_3_, ppm): *δ* 41.63, 123.3, 127.8, 128.6, 128.7, 132.2, 134.0, 136.4, 168.0; HRMS (ESI) calcd for C_8_H_8_OS [M + H]^+^ 238.0863; found 238.0862.

##### (1,3-Dioxoisoindolin2-yl) (phenyl)methyl acetate (q)^23^

2-Benzylisoindoline-1,3-dione (1.5 g, 6.3 mol) was dissolved in 25 mL of chlorobenzene. *N*-Bromosuccinamide (1.68 g, 9.4 mol), sodium acetate (0.77 g, 9.4 mol) and acetic acid (0.54 mL, 9.4 mol) was added to above reaction mixture. Then reaction mixture was refluxed with constant stirring for 12 h. After completion of reaction, reaction mixture evaporated on reduced pressure then extracted with ethyl acetate (3 × 15 mL). Combined organic layer wash with brine and dried over Na_2_SO_4_. Crude product was purified by column chromatography using silica gel (100–200 mesh) and 10% ethyl acetate/pet. ether as eluent. Desired product as a white solid in 75% yield. (30 mg); ^1^H NMR (200 MHz, CDCl_3_, ppm): *δ* 2.2 (s, 3H), 7.4 (m, 3H), 7.6 (d, *J* = 7.25 Hz, 2H), 7.7 (s, 1H), 7.8 (m, 2H), 7.9 (m, 2H); ^13^C NMR (50 MHz, CDCl_3_, ppm): *δ* 20.8, 74.2, 123.8, 126.4, 128.5, 129.0, 131.6, 134.5, 135.1, 166.3, 169.3; HRMS (ESI) calcd for C_8_H_8_OS [M + Na]^+^ 318.0737; found 318.0733.

##### Amino(phenyl)methyl acetate (r)^23^

(1,3-dioxoisoindolin2-yl)-(phenyl)methyl acetate (1 g, 3.38 mol) was taken in two neck round bottom flasks and 20 mL of ethanol was added. To above solution hydrazine hydrate (0.17 g, 3.38 mol) was added and refluxed for 20 minutes. After 20 minutes acetic acid (0.58 mL, 5.1 mol) was added and further refluxed for 2 h. Then EtOH was evaporated on reduced pressure and extracted with ethyl acetate (3 × 15 mL). Combined organic layer wash with brine and dried over Na_2_SO_4_. Crude product was purified using silica gel (100–200 mesh) and 10% ethyl acetate/pet. ether as eluent. Desired product was obtained as white solid in 60% yield (30 mg). ^1^H NMR (200 MHz, CDCl_3_, ppm): *δ* 2.4 (s, 3H), 7.4 (m, 3H), 7.7 (dd, *J* = 7.06, 2.48 Hz, 2H), 7.9 (s, 1H), 10.2 (s, 1H); ^13^C NMR (50 MHz, CDCl_3_, ppm): *δ* 20.3, 127.1, 128.7, 130.0, 133.9, 143.7, 174.0; HRMS (ESI) calcd for C_8_H_8_OS [M + Na]^+^ 188.0682; found 188.2123.

## Conclusions

In conclusion, we have developed a rapid and metal-free oxidation protocol to access carbonyl compounds from primary and secondary amines using PhI(OAc)_2_ in combination with catalytic amount of TEMPO as an eco-friendly oxidation without the need of external oxygen source under mild conditions. In addition, we established the mechanistic pathway, with aid of control experiments, time-dependent ^1^H NMR and GC-MS analyses.

## Conflicts of interest

There are no conflicts to declare.

## Supplementary Material

RA-008-C8RA07451H-s001
