# Supplementary material for: Metal-free hypervalent iodine/TEMPO mediated oxidation of amines and mechanistic insight into the reaction pathways
Source: RSC Adv. 2018 Sep 14;8(56):32055–62. doi: 10.1039/c8ra07451h (PMC9086218; doi:10.1039/c8ra07451h)
Supplement: RA-008-C8RA07451H-s001 [file RA-008-C8RA07451H-s001.pdf]

Electronic Supplementary Material (ESI) for RSC Advances.
This journal is © The Royal Society of Chemistry 2018

Supporting information

Metal-Free Hypervalent Iodine/TEMPO Mediated Oxidation of Amines and Mechanistic Insight into the Reaction Pathways

Ajay H. Bansode[†‡] and Gurunath Surayvanshi*[†‡]

[†]*Chemical Engineering & Process Development Division, CSIR-National Chemical Laboratory,*

Dr. Homi Bhabha Road, Pune, Maharashtra, India- 411008.

[‡]*Academy of Scientific and Innovative Research (AcSIR), New Delhi 110 025, India*

*Corresponding author: Tel.: +91 20 25902547, Fax: +91 20 25902676

e-mail: gm.suryavanshi@ncl.res.in

Table of Contents:

**S. No.**	**Contents**	**Page No.**
1	GC-MS Data	S2-S3
2	^{1}H and ^{13}C NMR spectra	S4-S31

Further Mechanistic Study by Using GC-MS of reaction mechanism

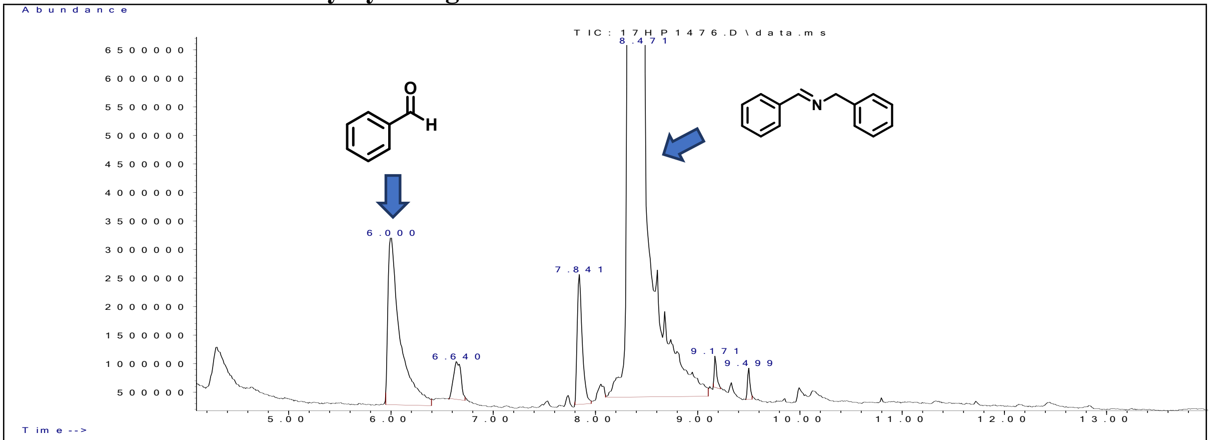

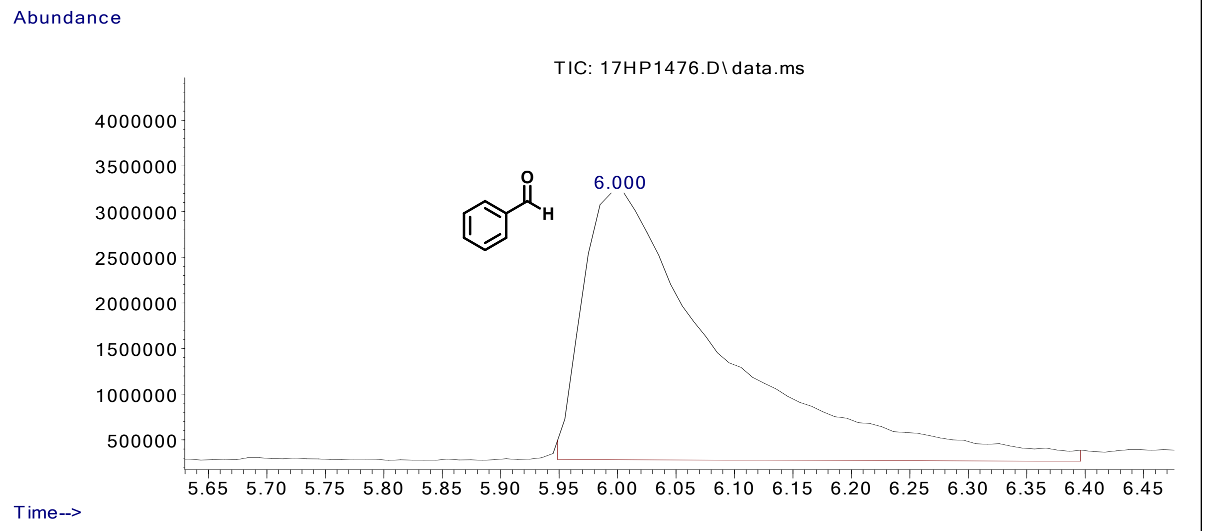

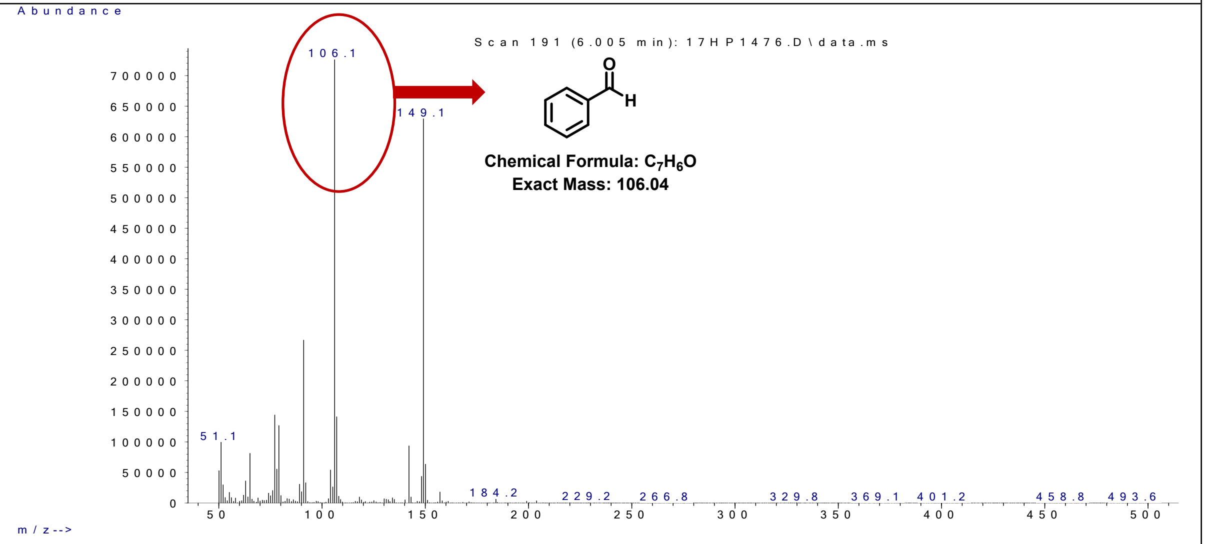

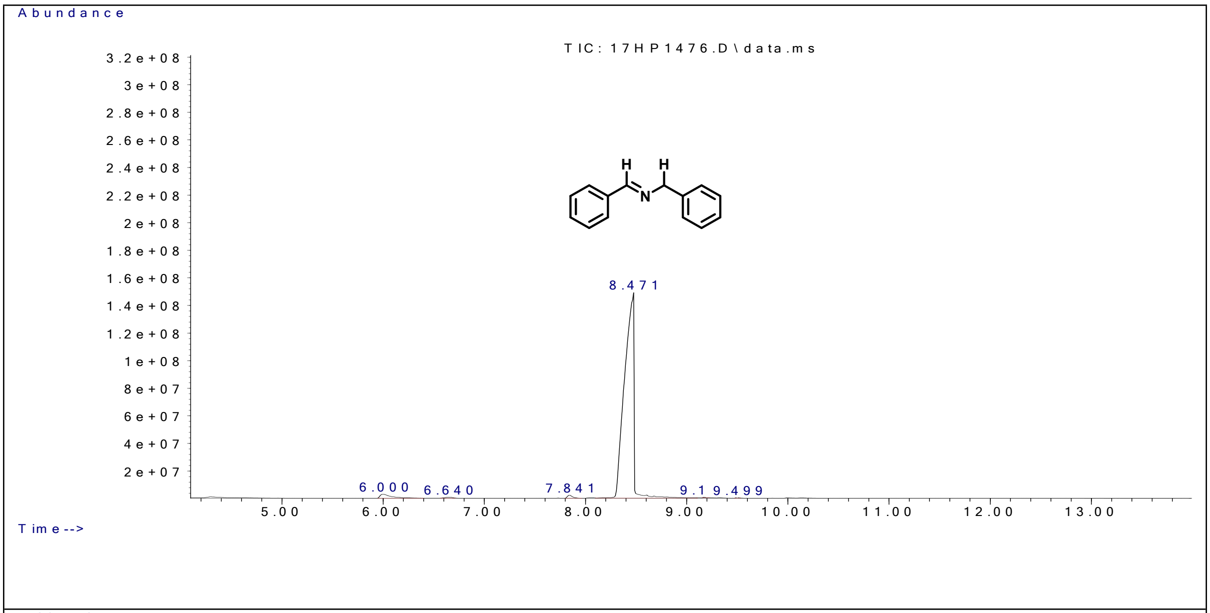

Abundance
TIC: 17HP1476.D\data.ms
8.471
6.000
6.640
7.841
9.1 9.499
Time-->

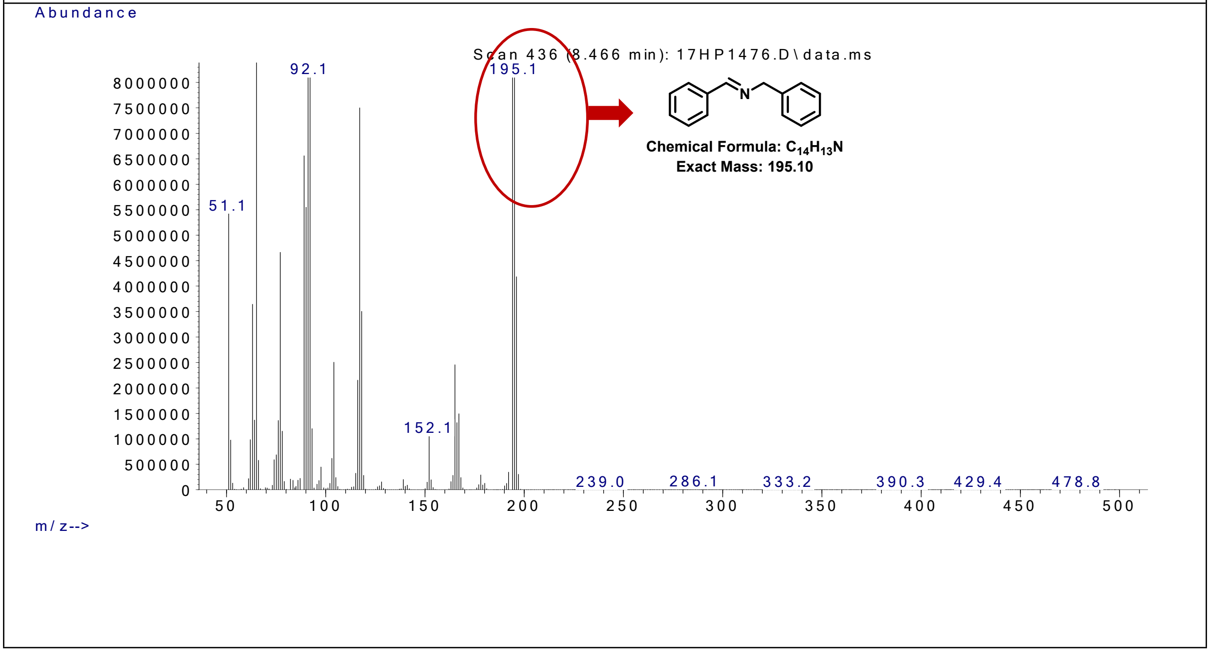

Abundance
Scan 436 (8.466 min): 17HP1476.D\data.ms
92.1
195.1
51.1
152.1
239.0
286.1
333.2
390.3
429.4
478.8
Chemical Formula: C14H13N
Exact Mass: 195.10
m/z-->

^{1}H and [13]C Spectra

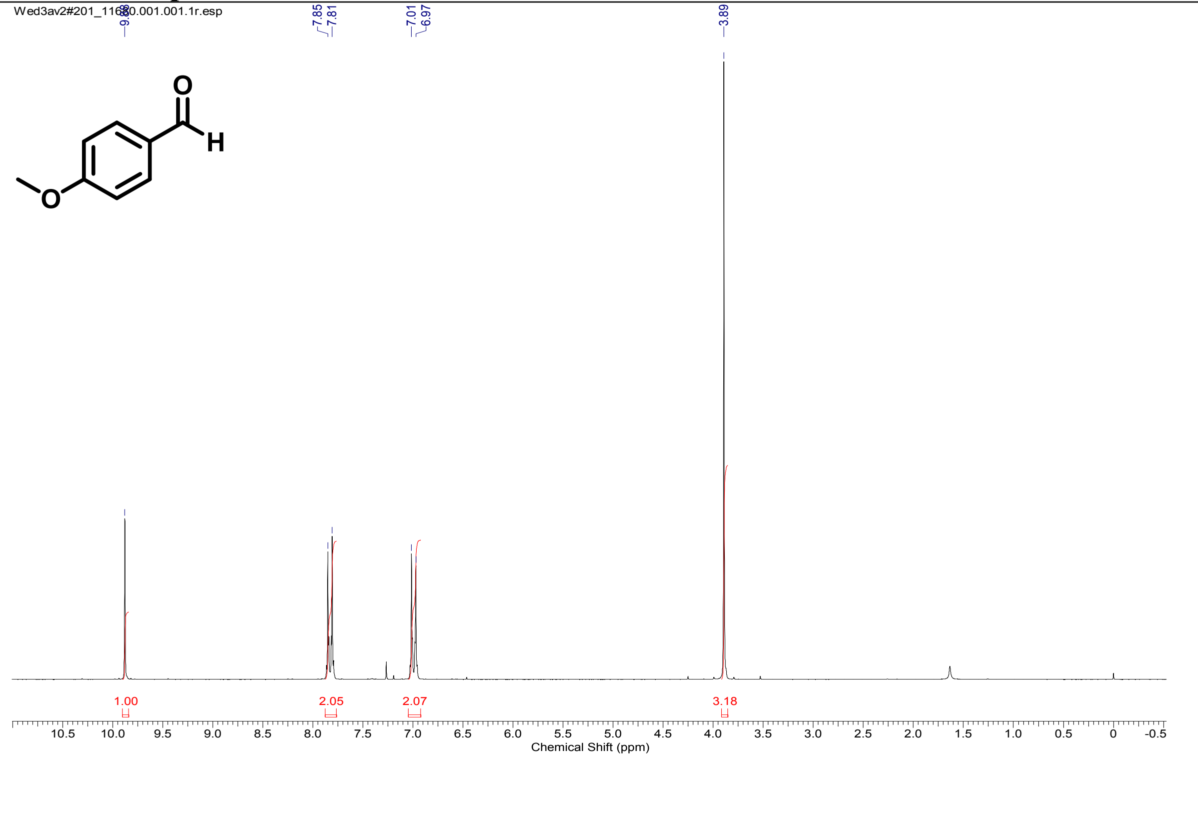

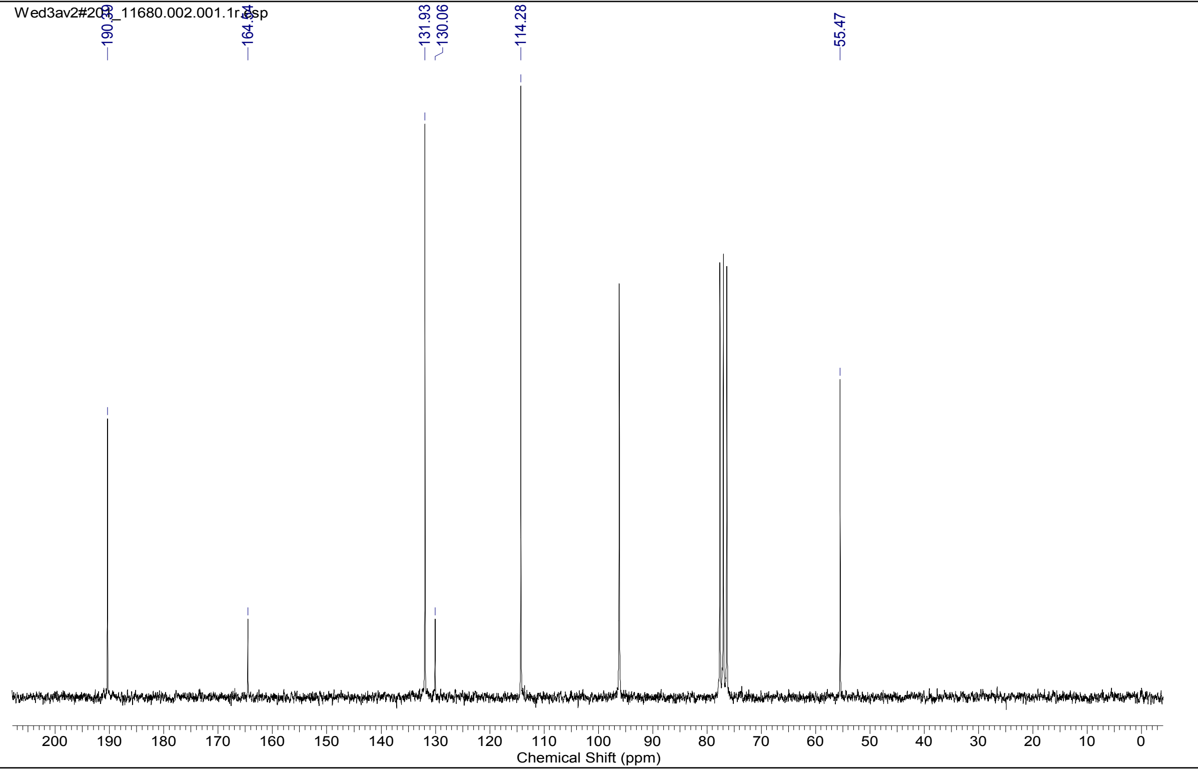

^{1}H and [13]C Spectra of 2a

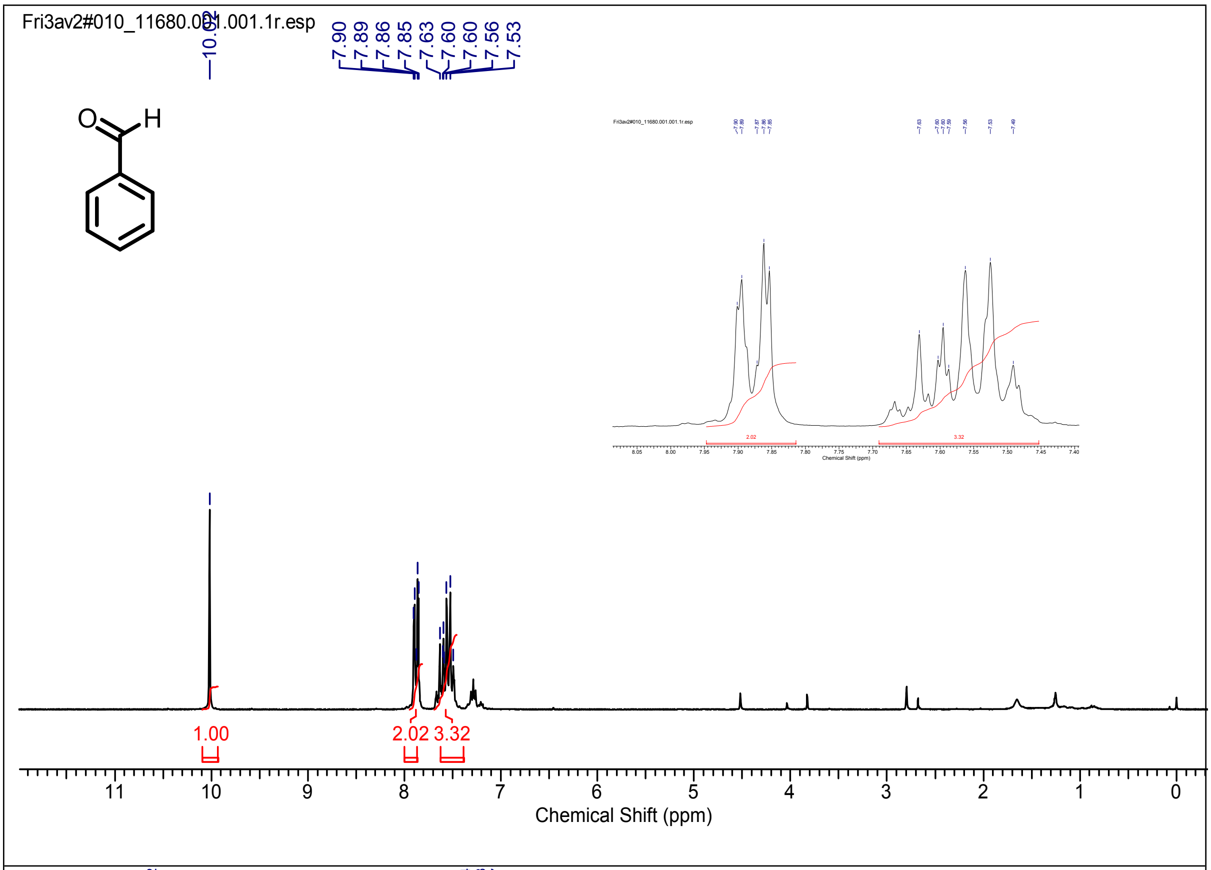

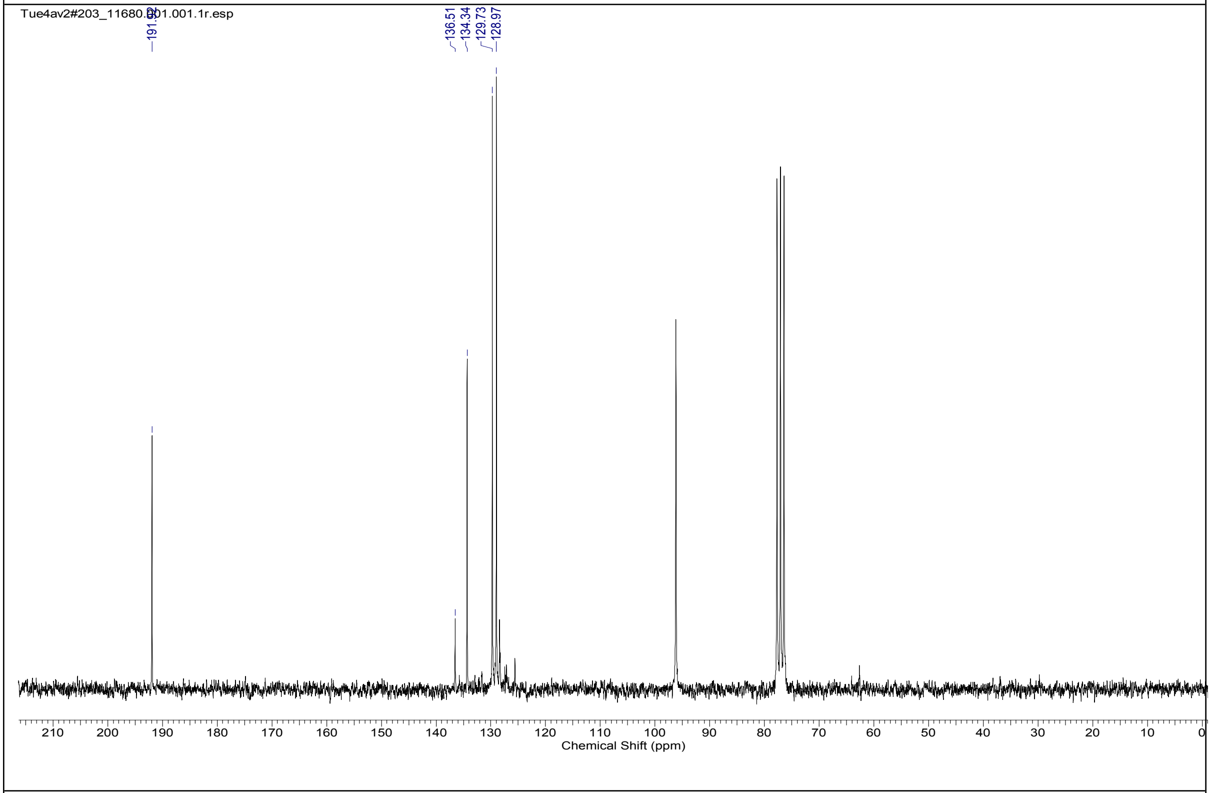

^{1}H and ^{13}C Spectra of 2b

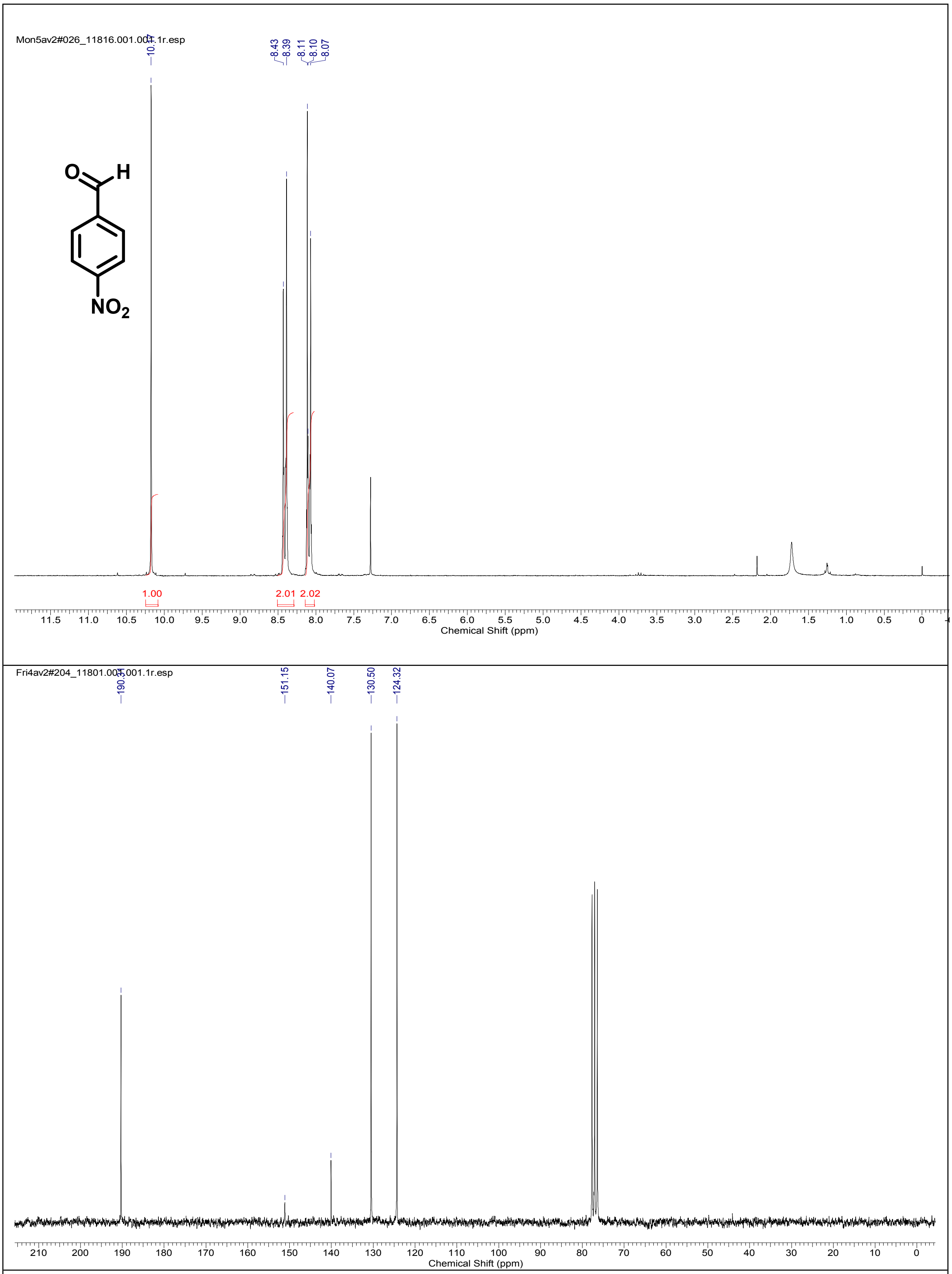

^{1}H and ^{13}C Spectra of 2c

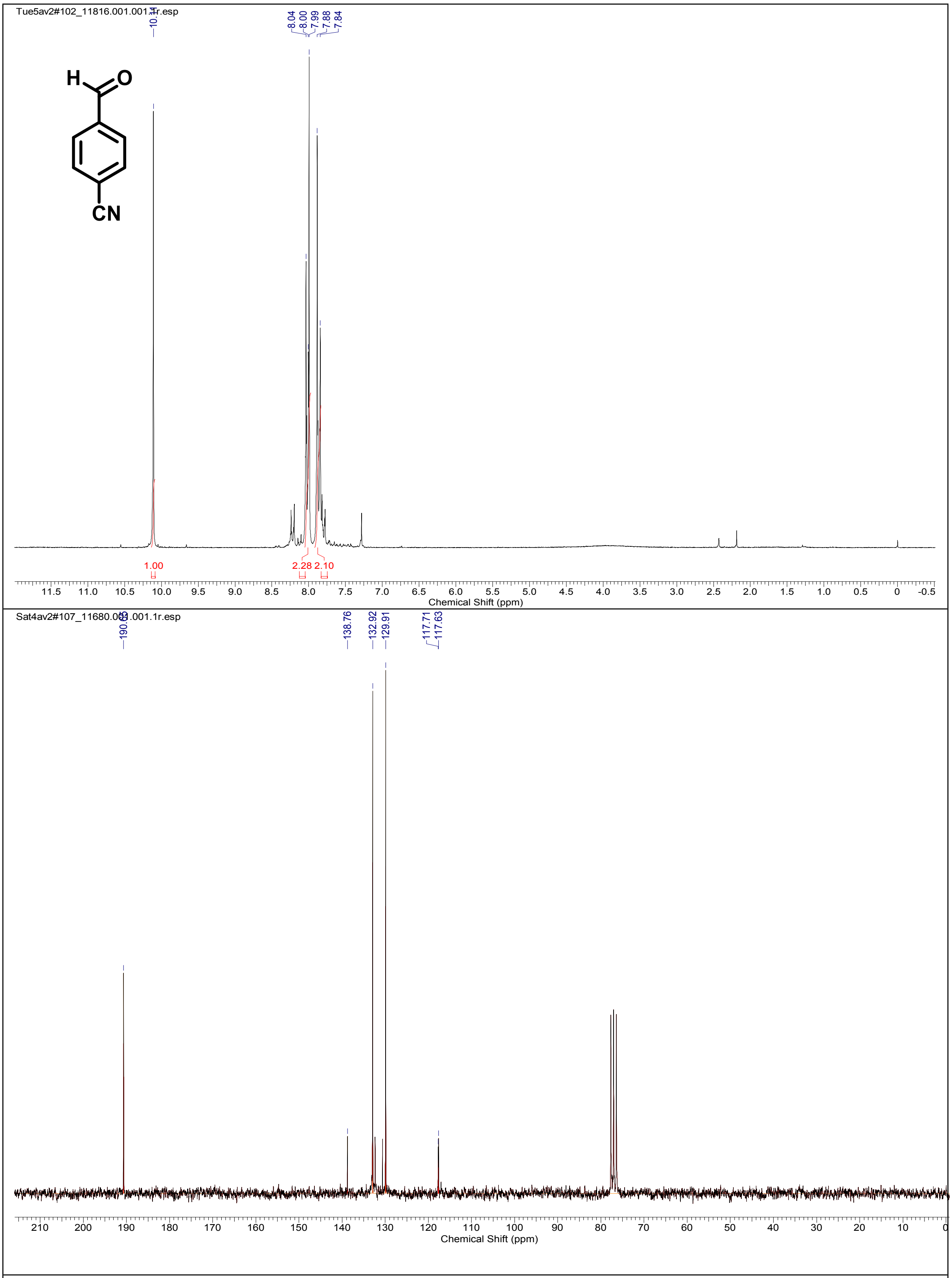

^{1}H and ^{13}C Spectra of 2d

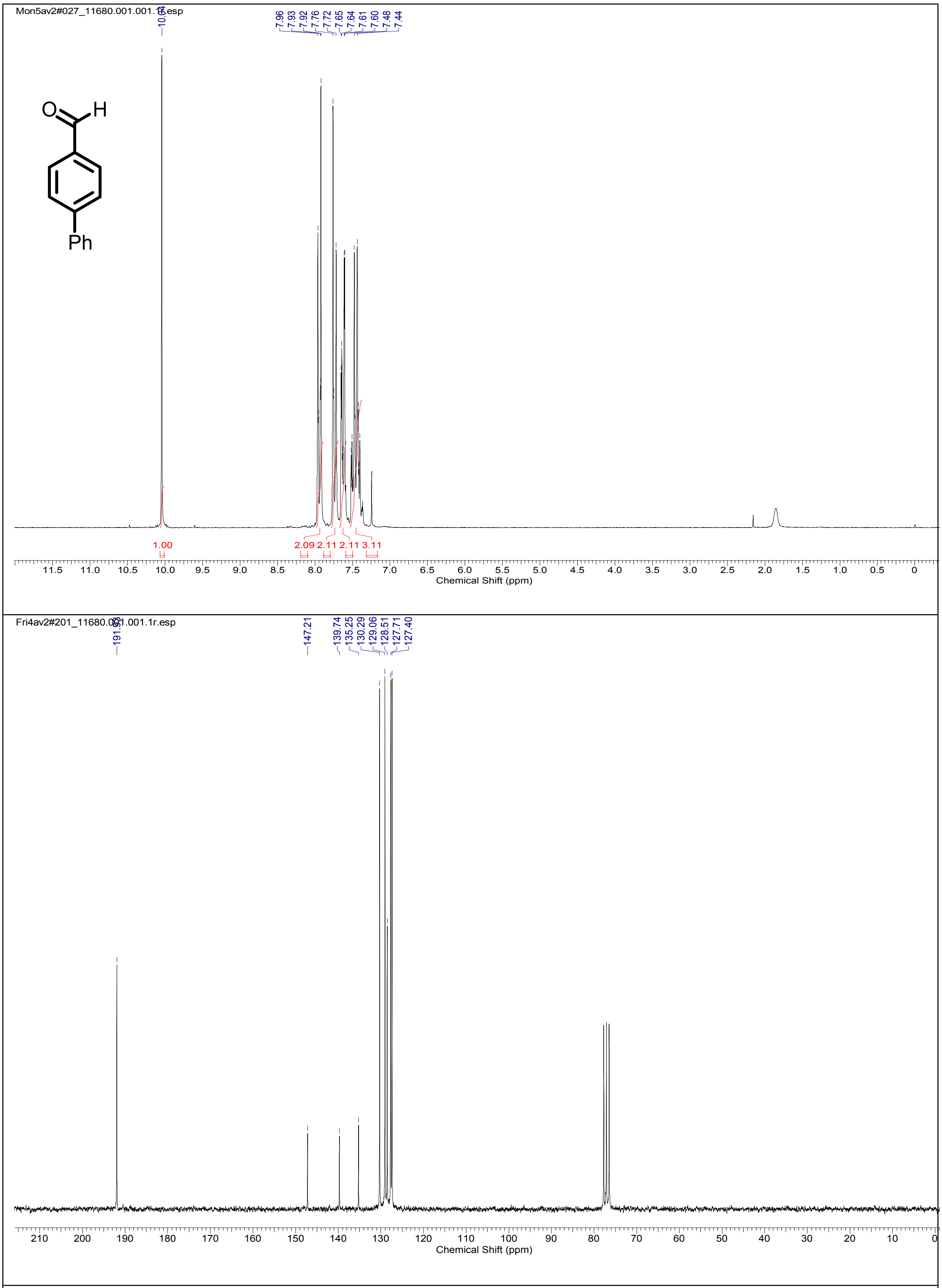

^{1}H and [13]C Spectra of 2e

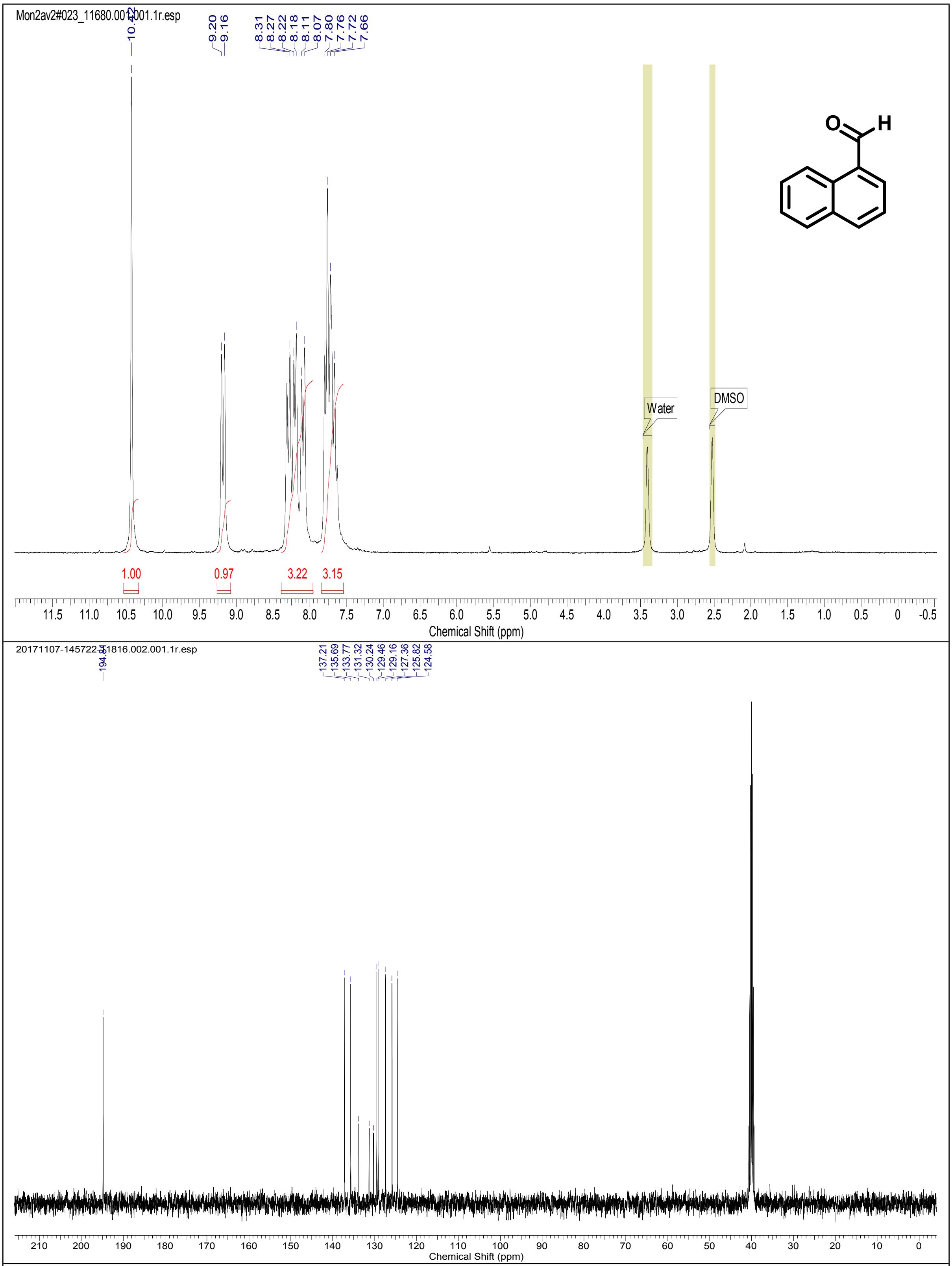

¹H and ¹³C Spectra of 2f

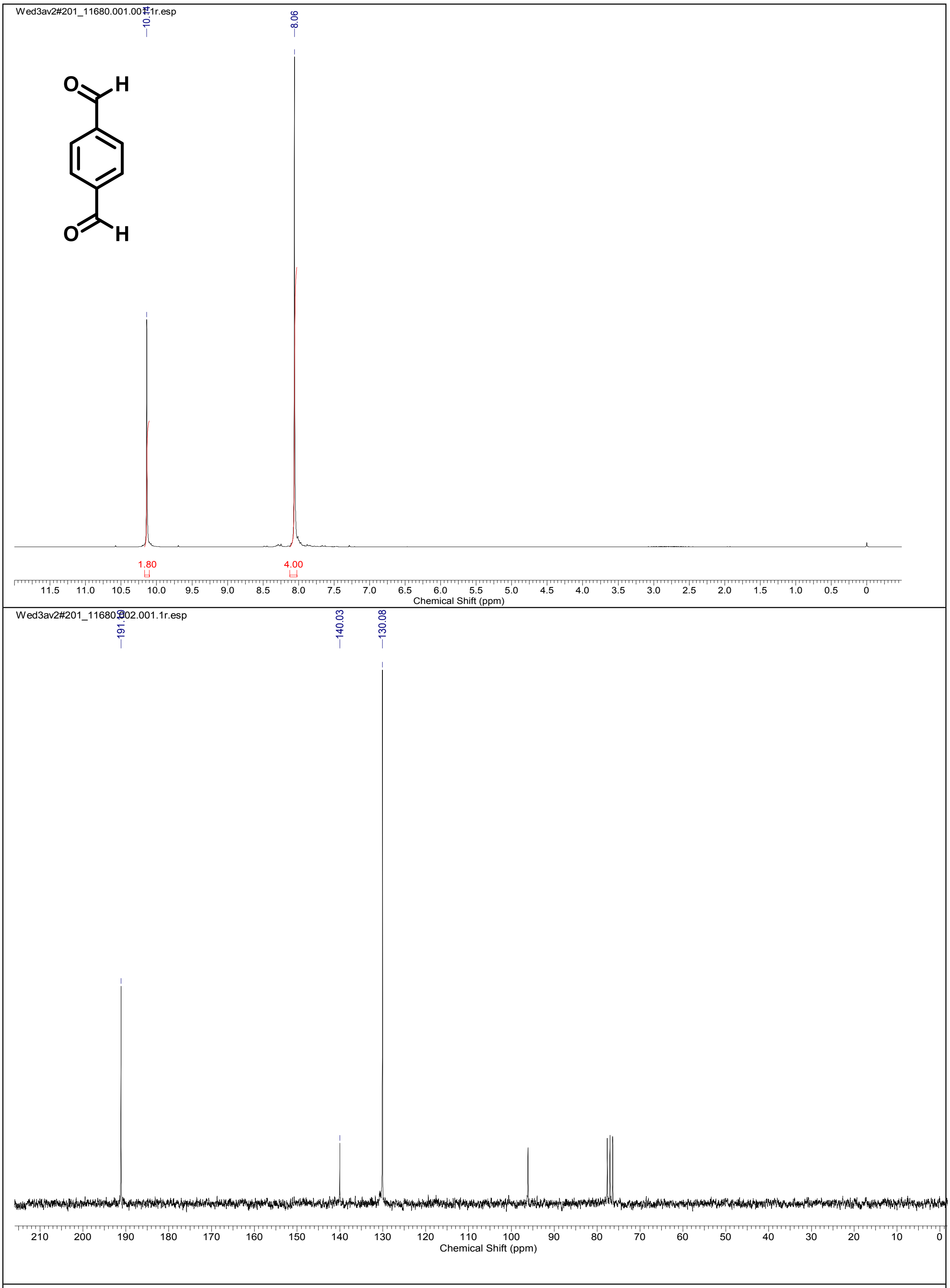

^{1}H and ^{13}C Spectra of 2g

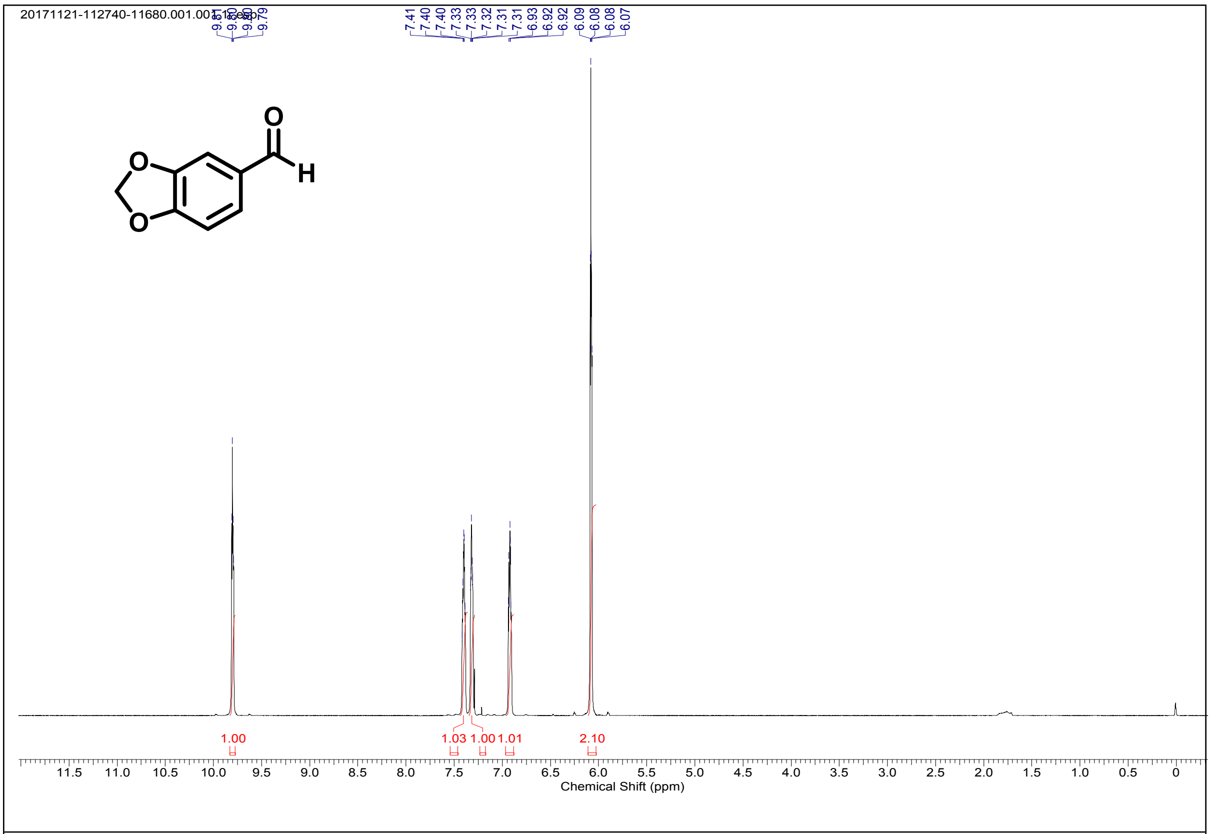

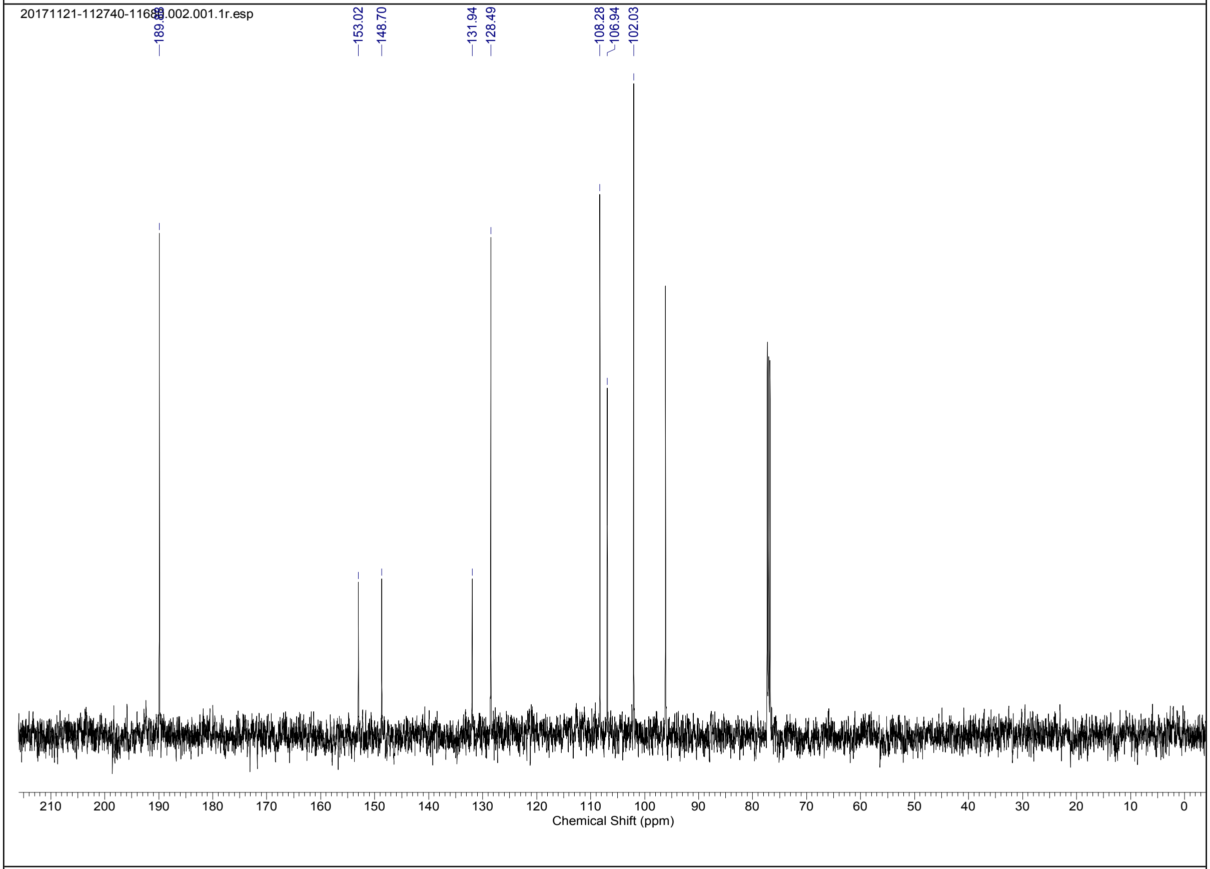

^{1}H and [13]C Spectra of 2h

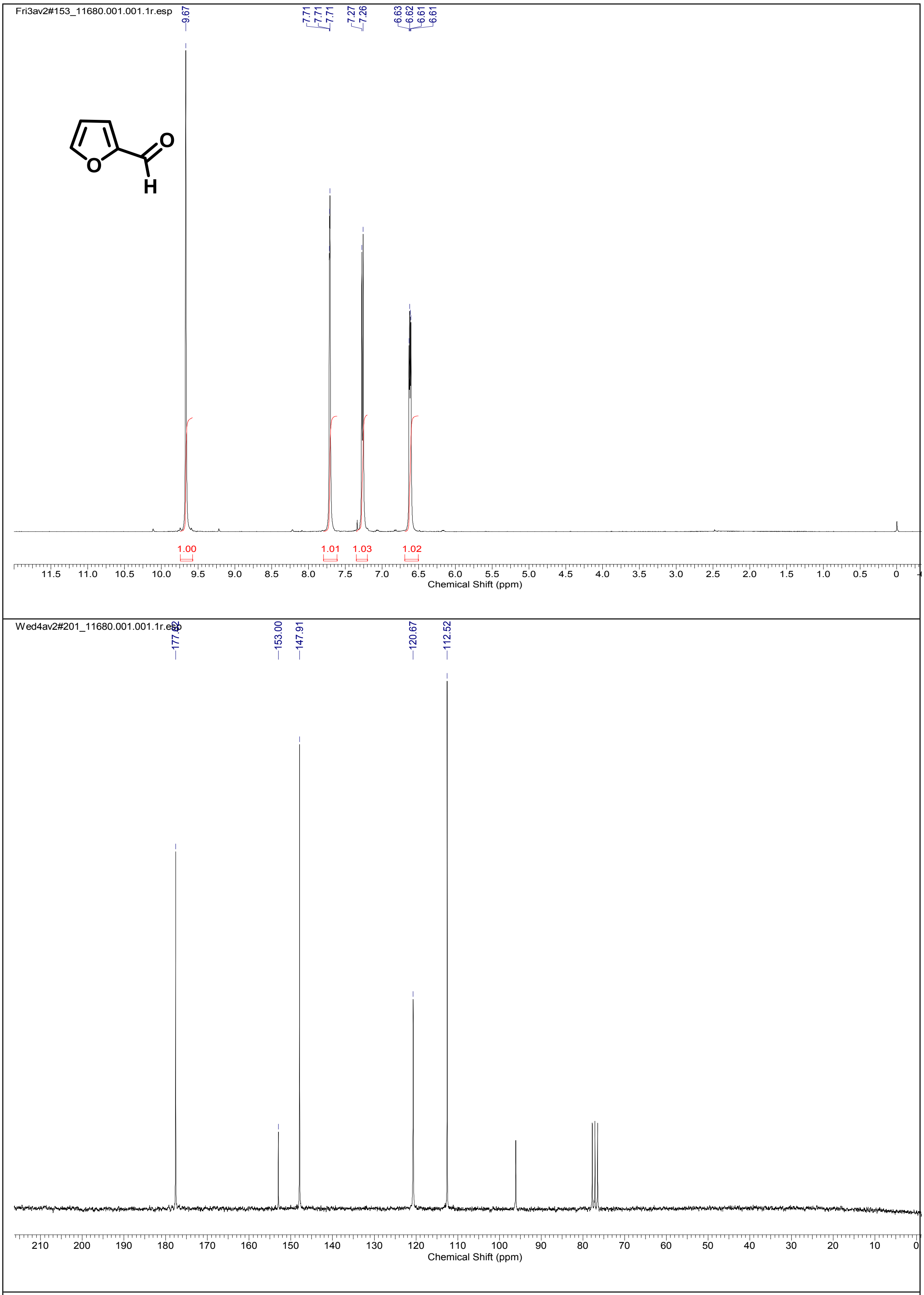

^{1}H and ^{13}C Spectra of 2i

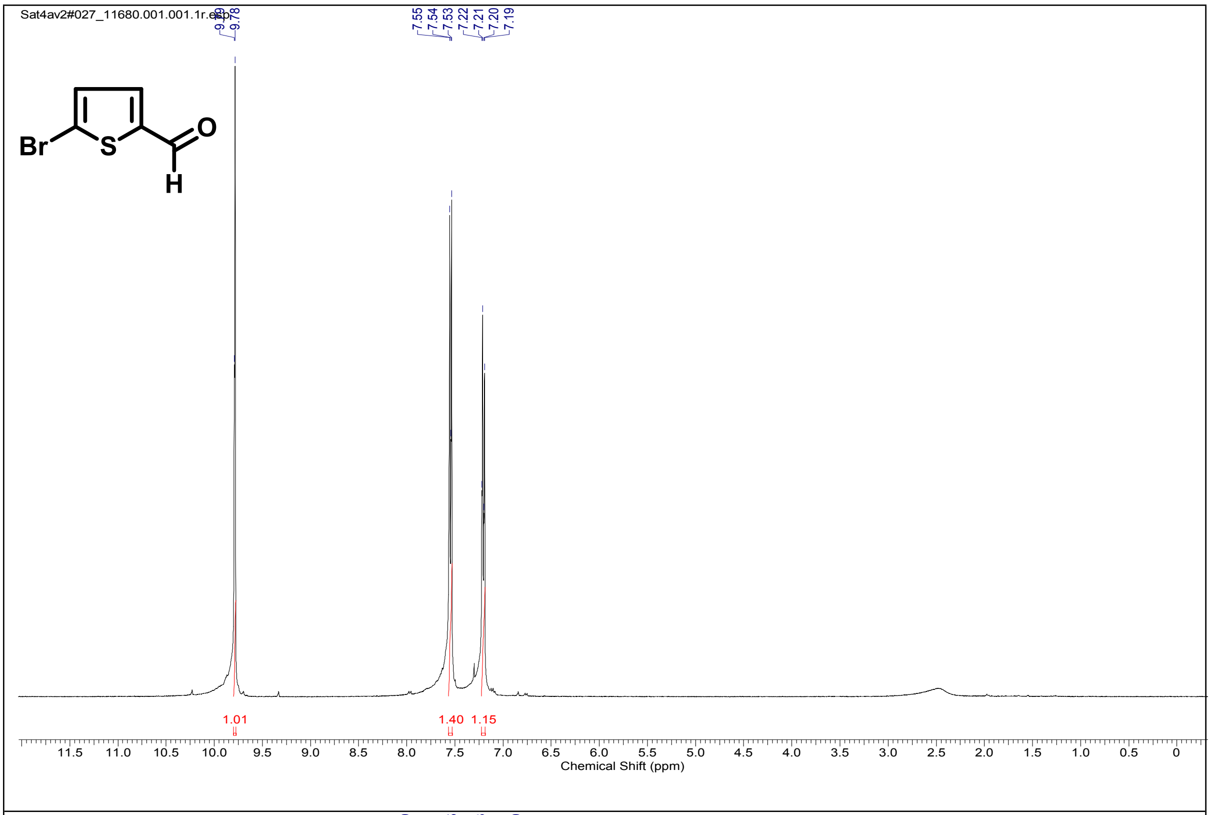

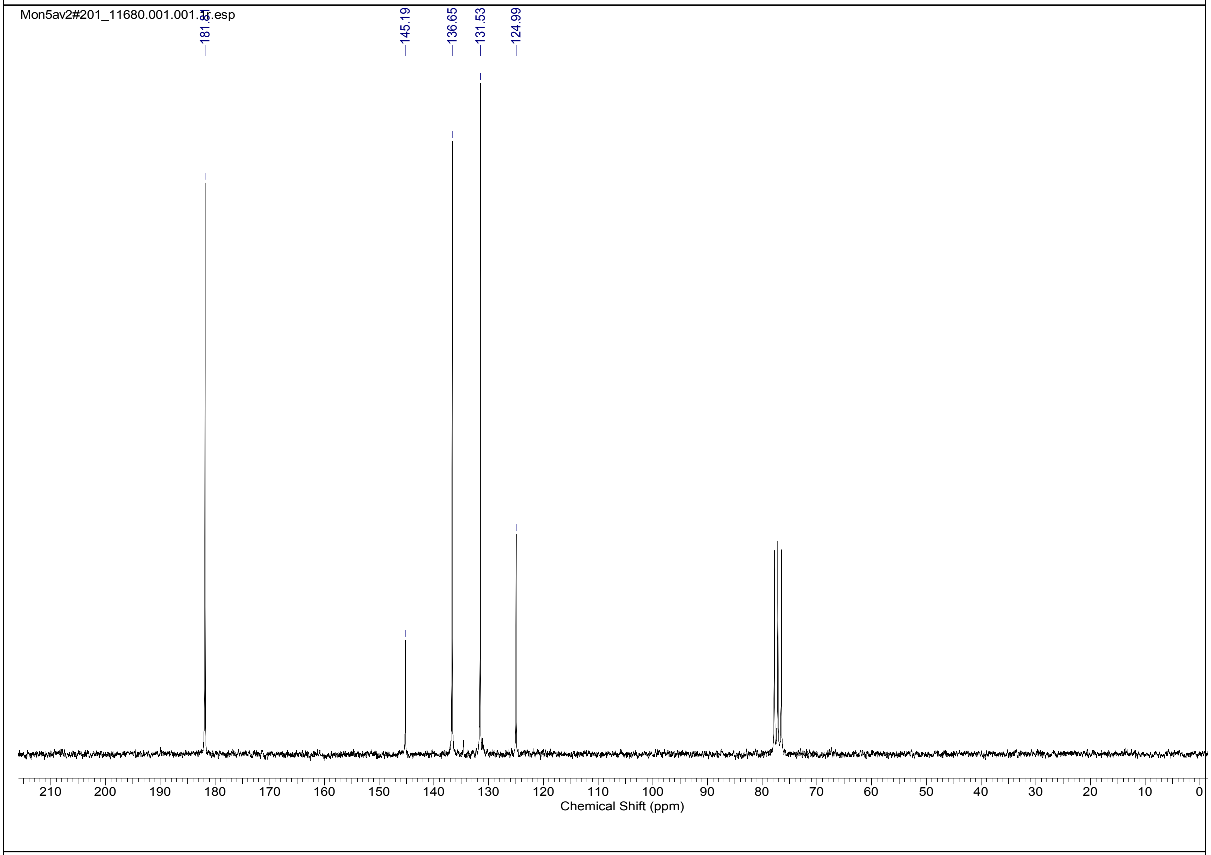

^{1}H and ^{13}C Spectra of 2j

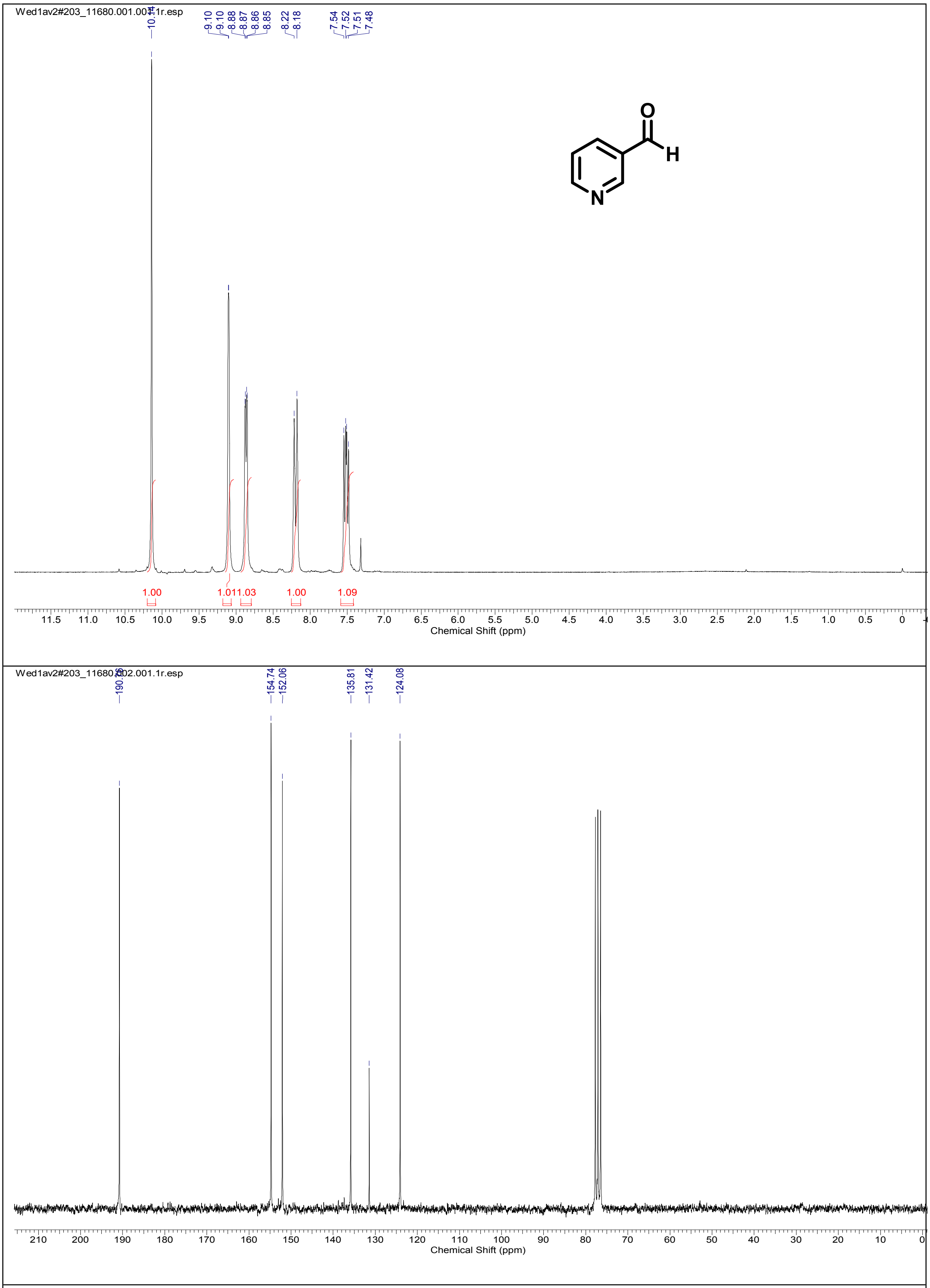

^{1}H and ^{13}C Spectra of 2k

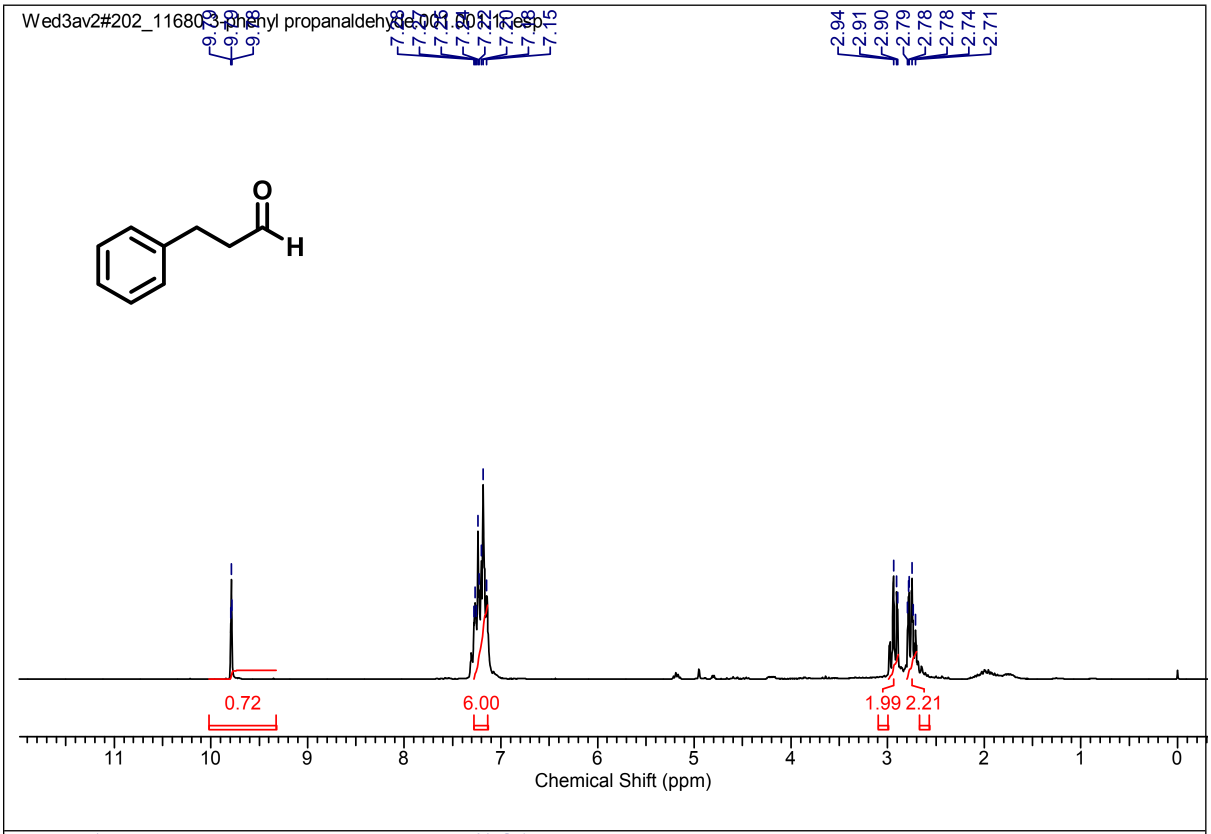

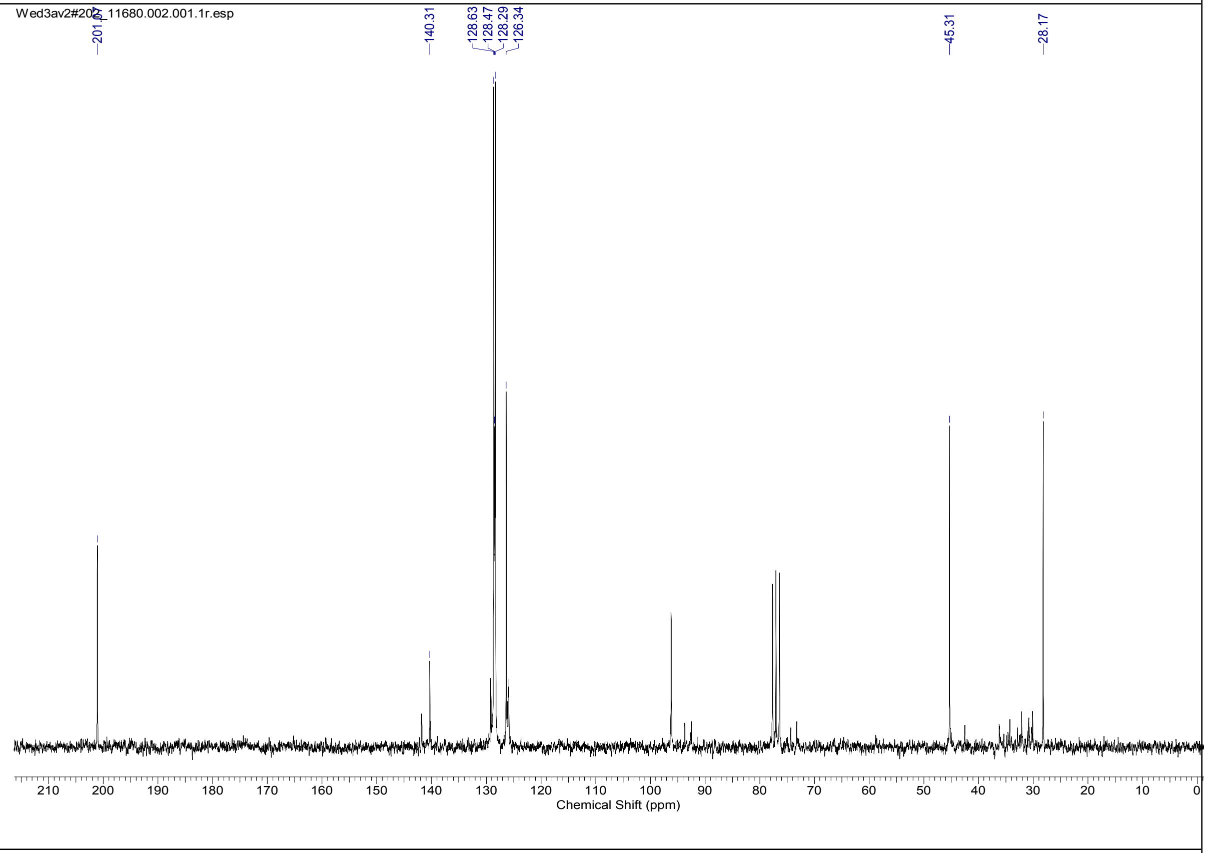

^{1}H and ^{13}C Spectra of 2l

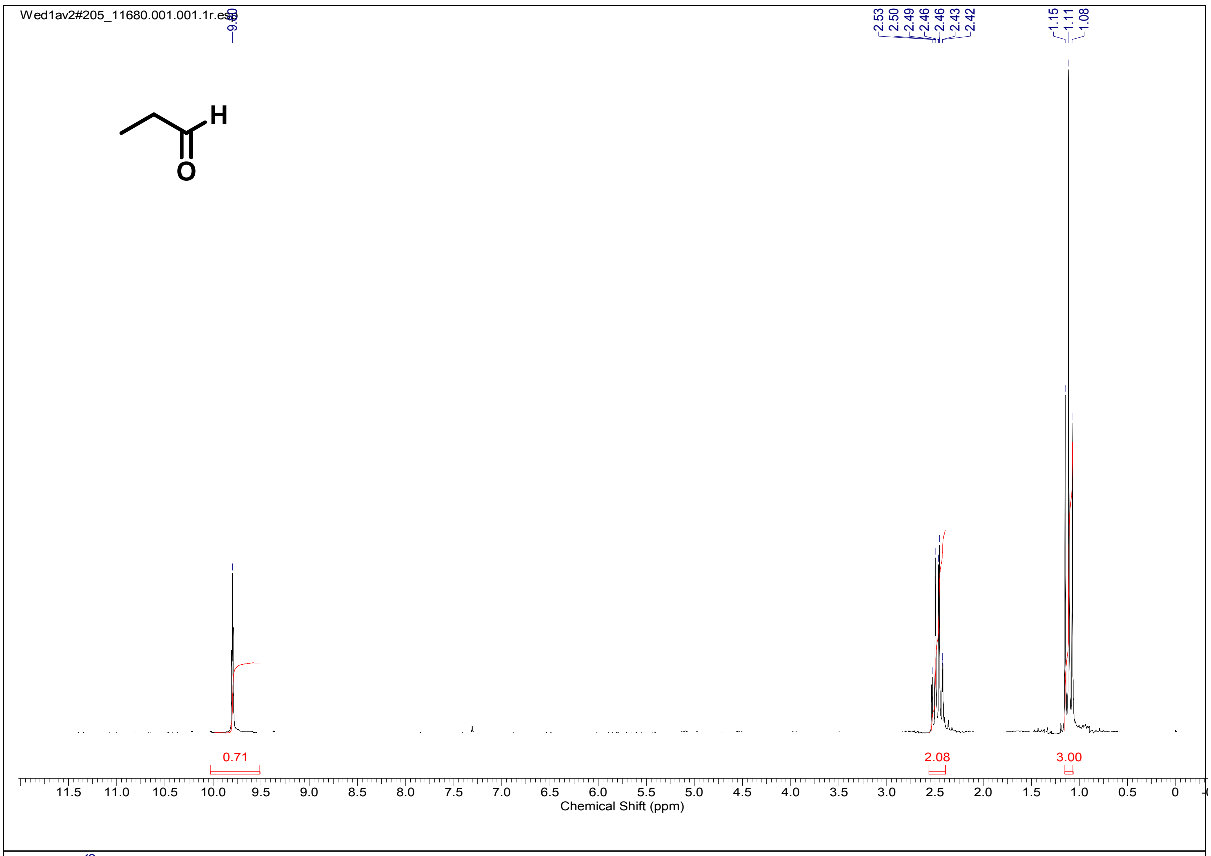

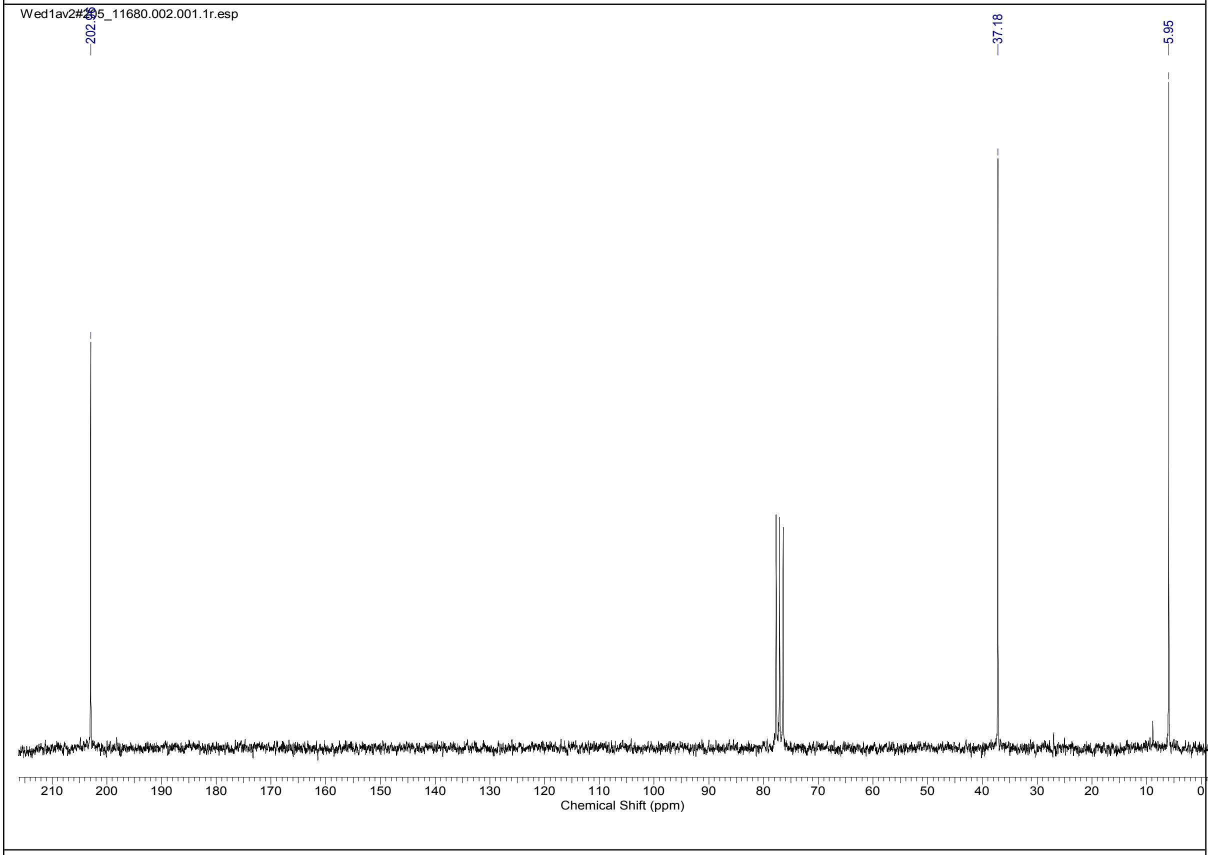

^{1}H and ^{13}C Spectra of 2m

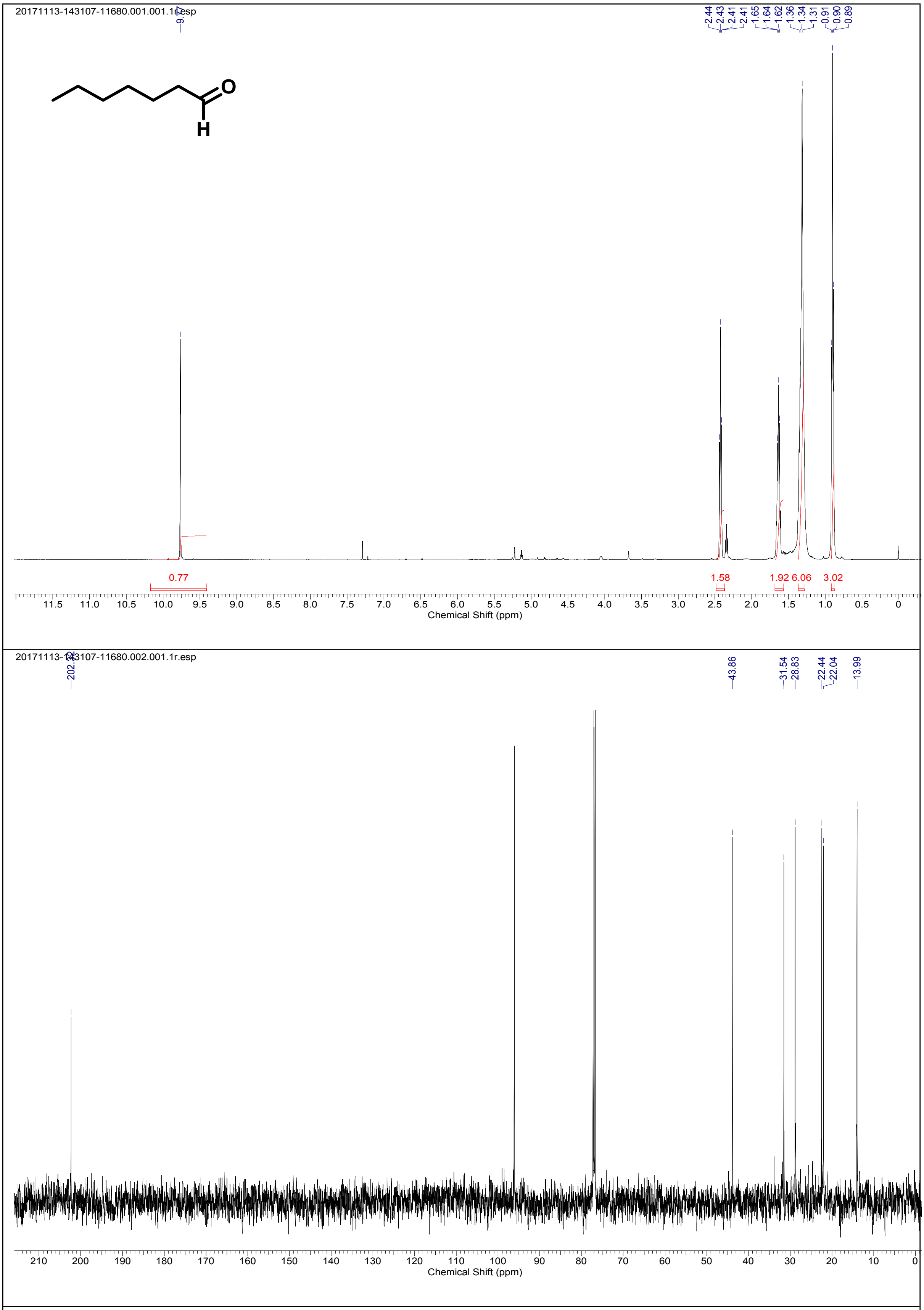

^{1}H and ^{13}C Spectra of 2n

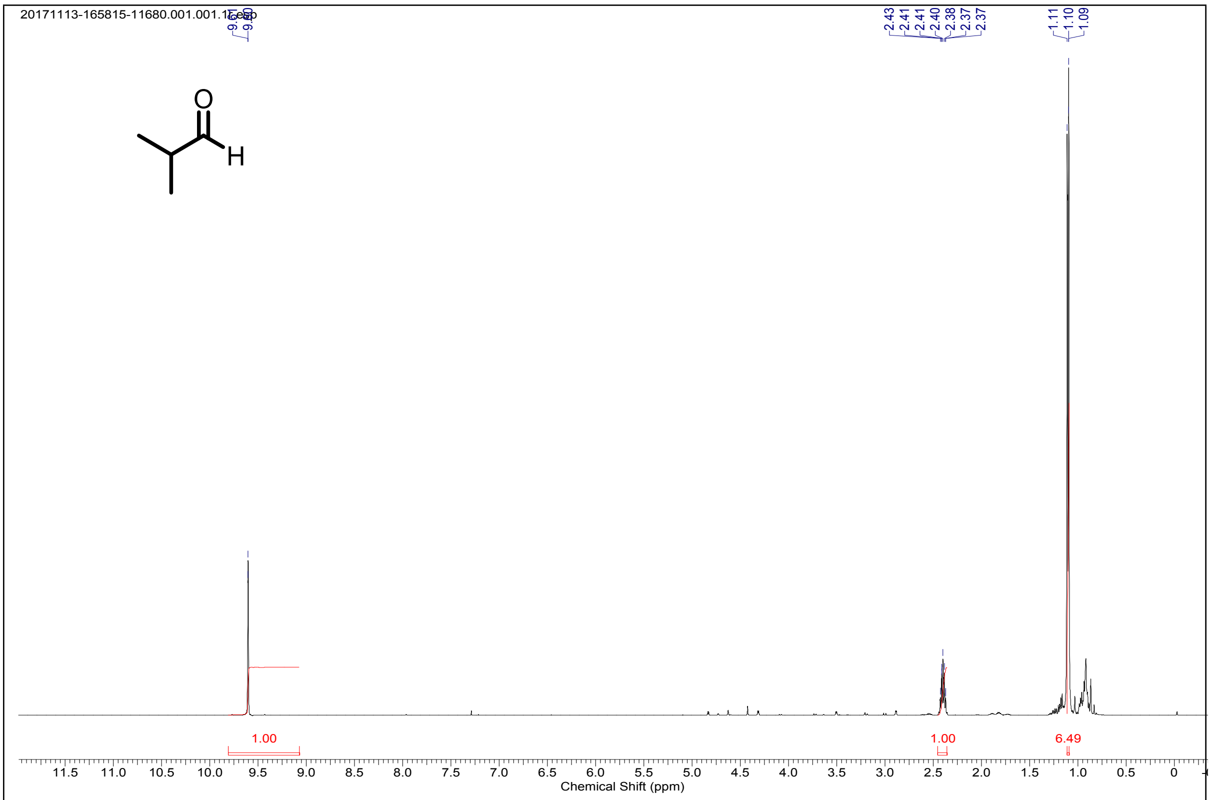

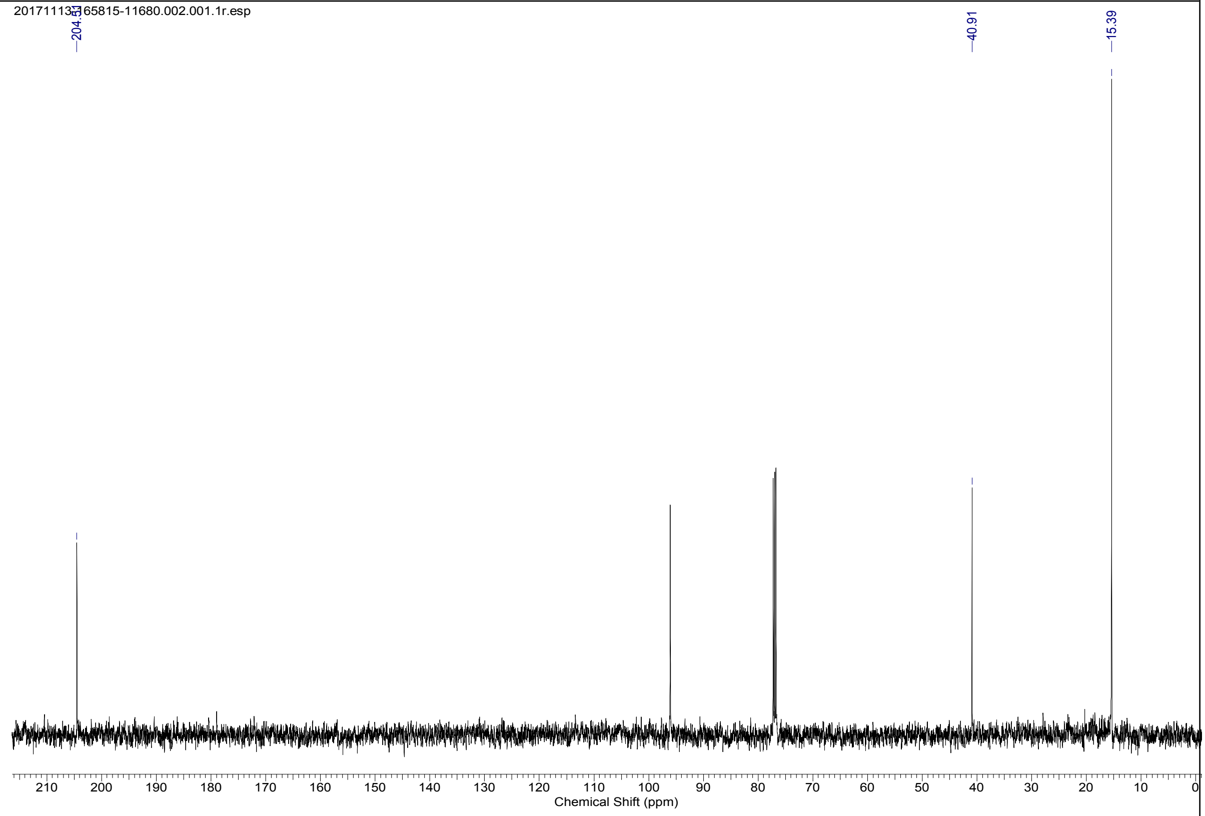

^{1}H and ^{13}C Spectra of 2o

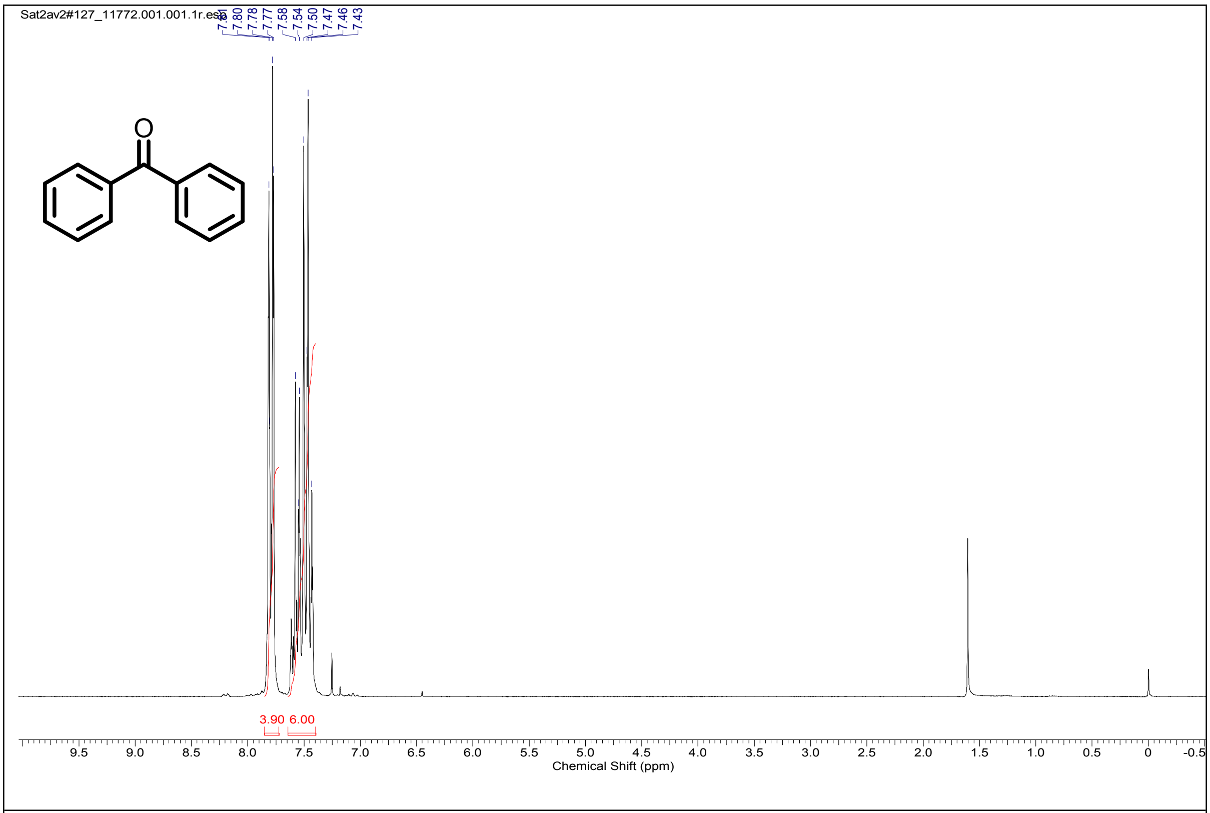

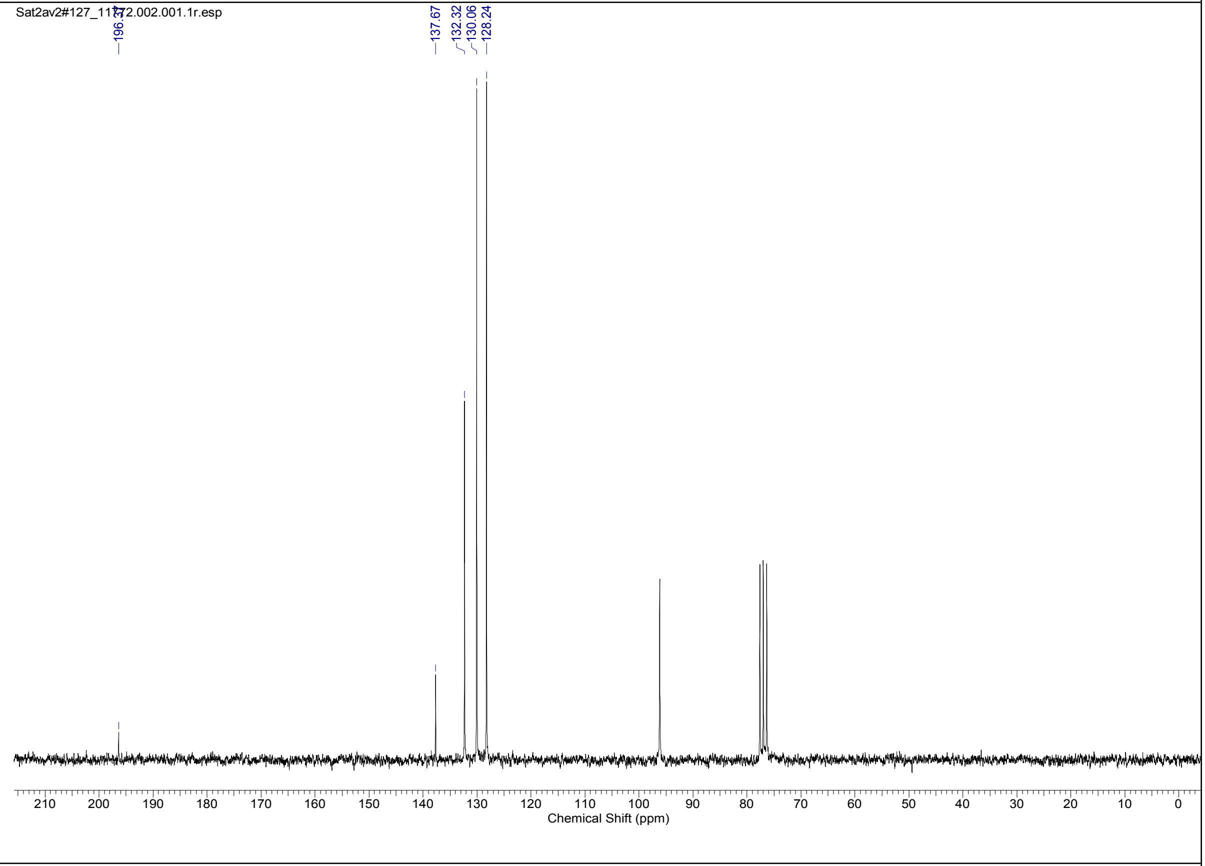

^{1}H and ^{13}C Spectra of 2p

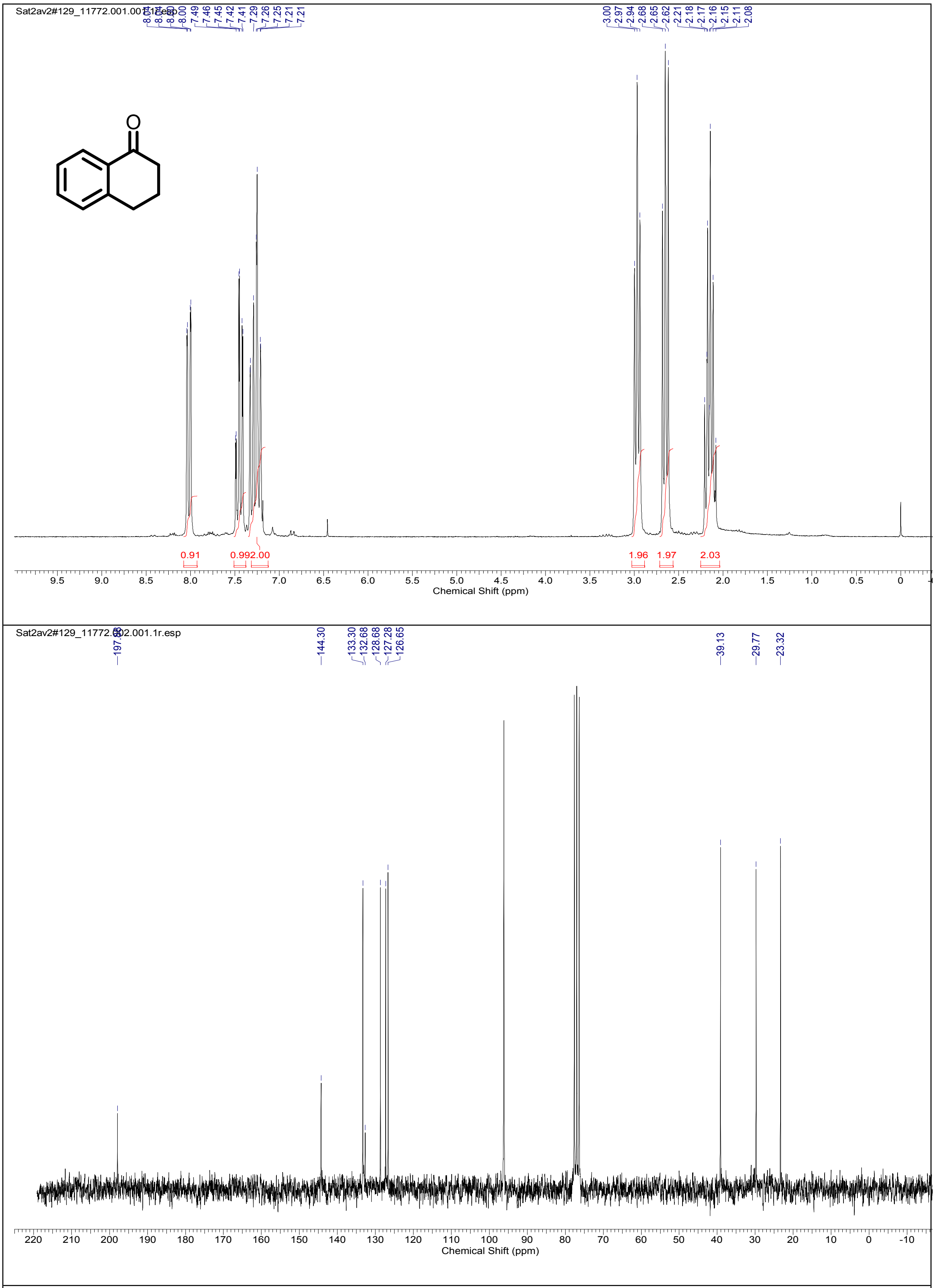

^{1}H and ^{13}C Spectra of 2q

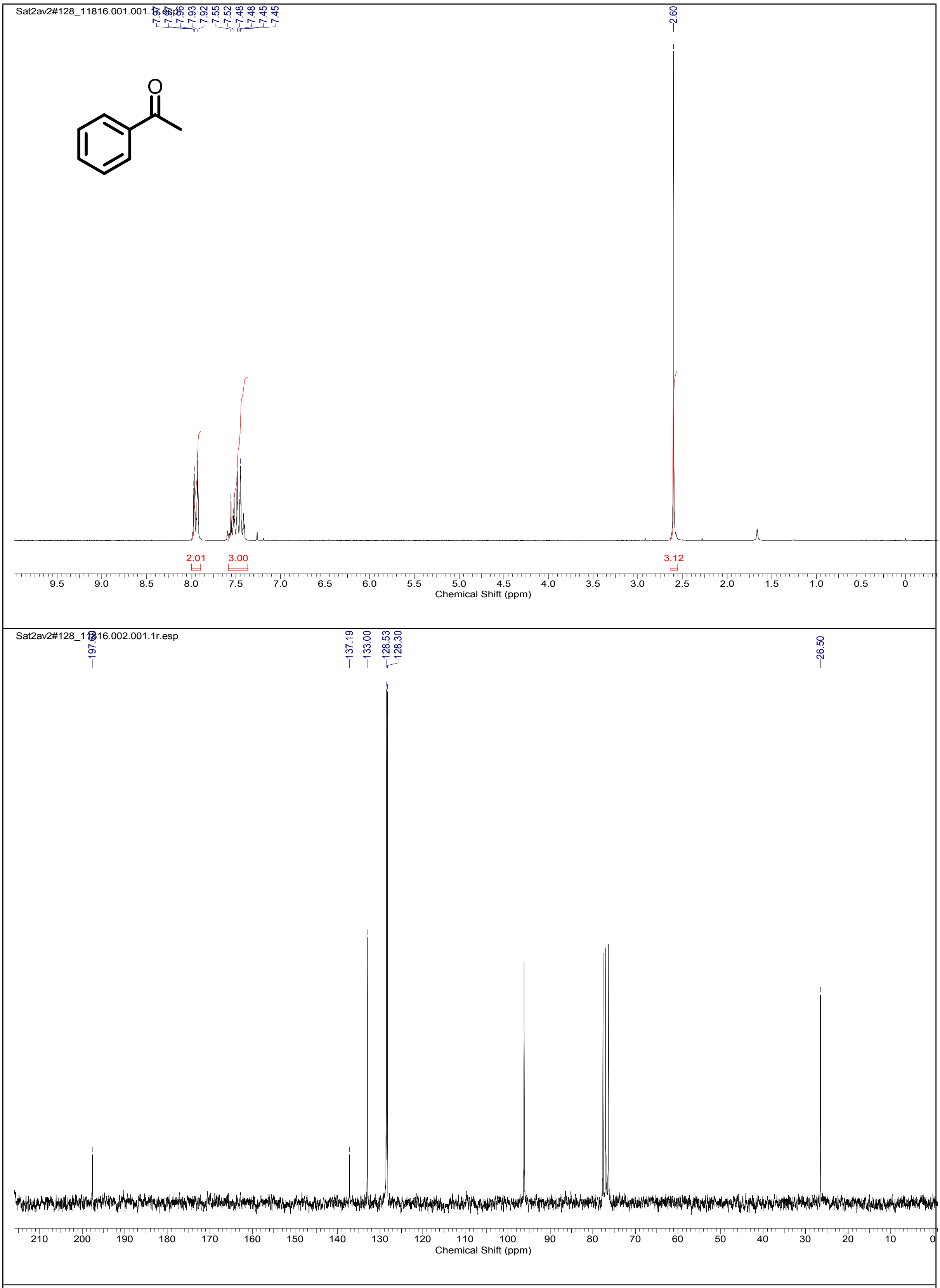

^{1}H and ^{13}C Spectra of 2r

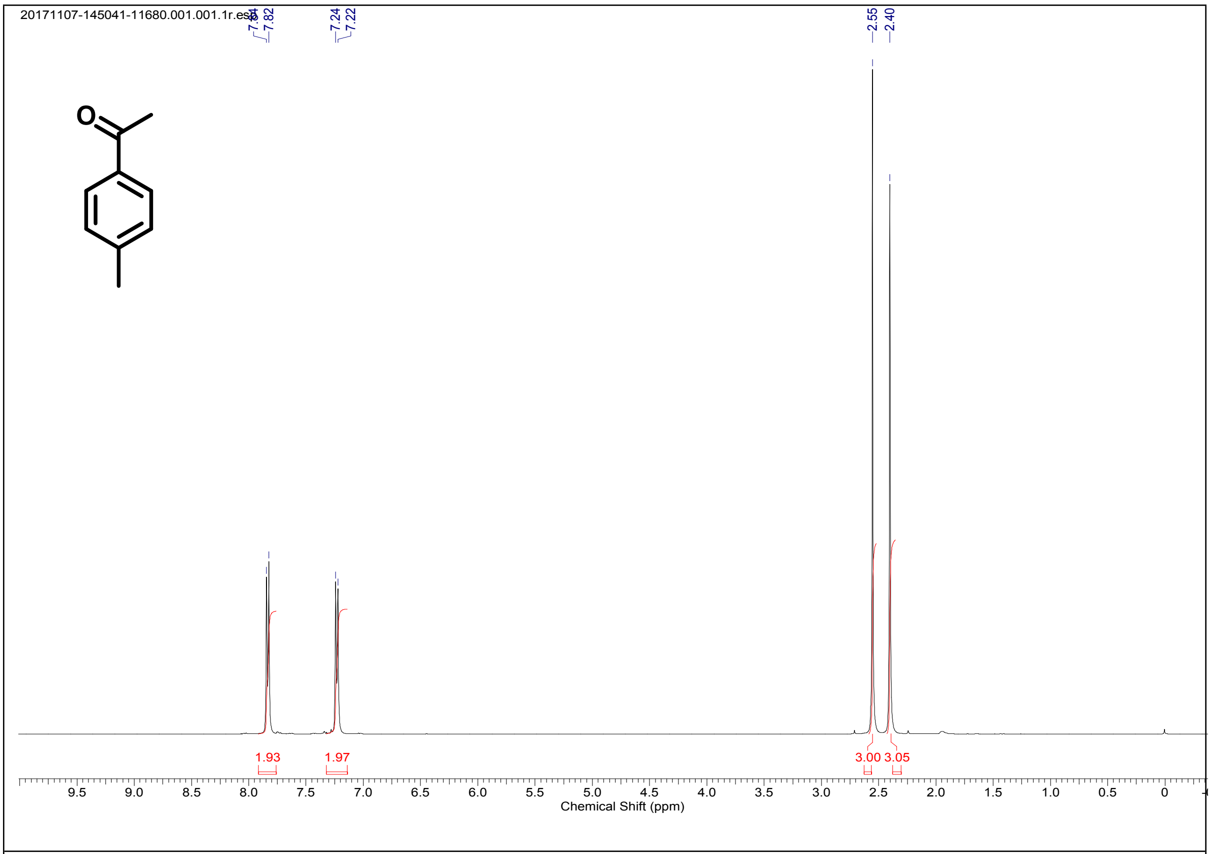

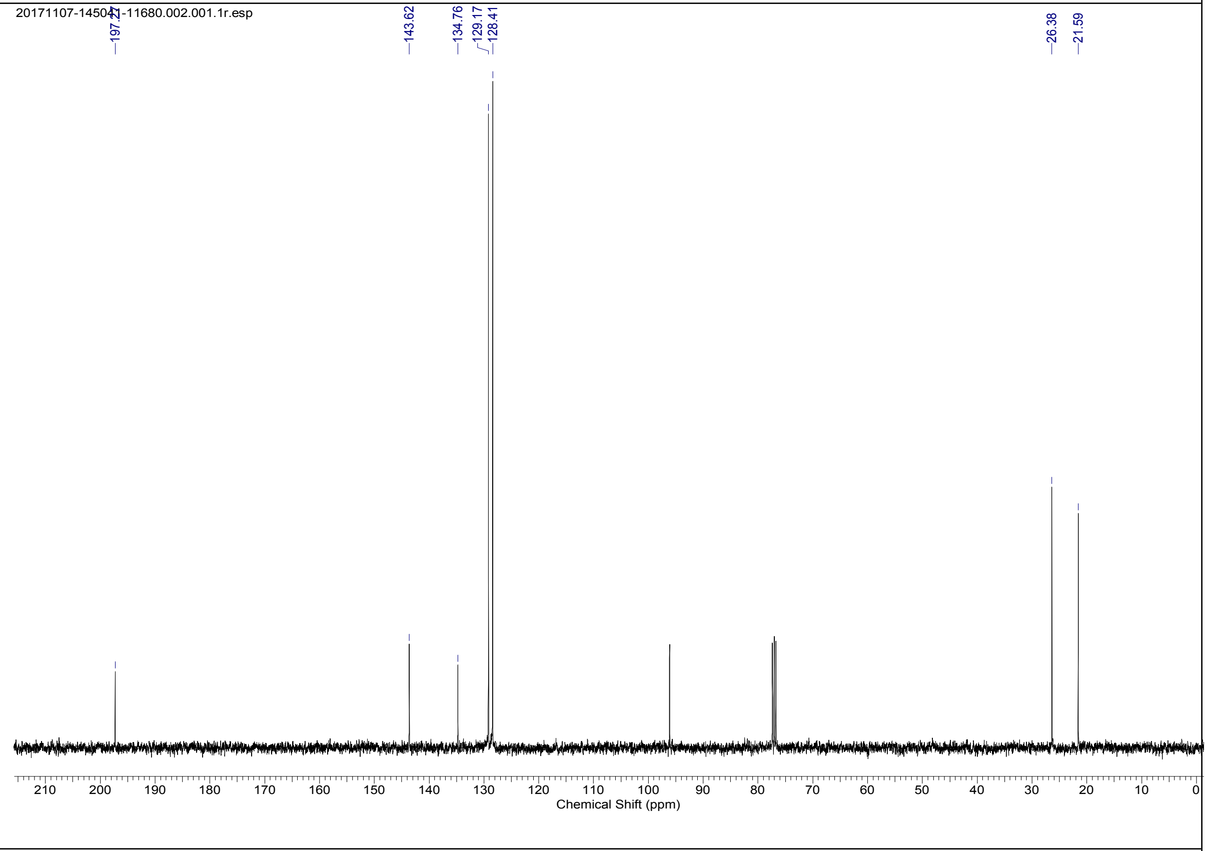

^{1}H and ^{13}C Spectra of 2s

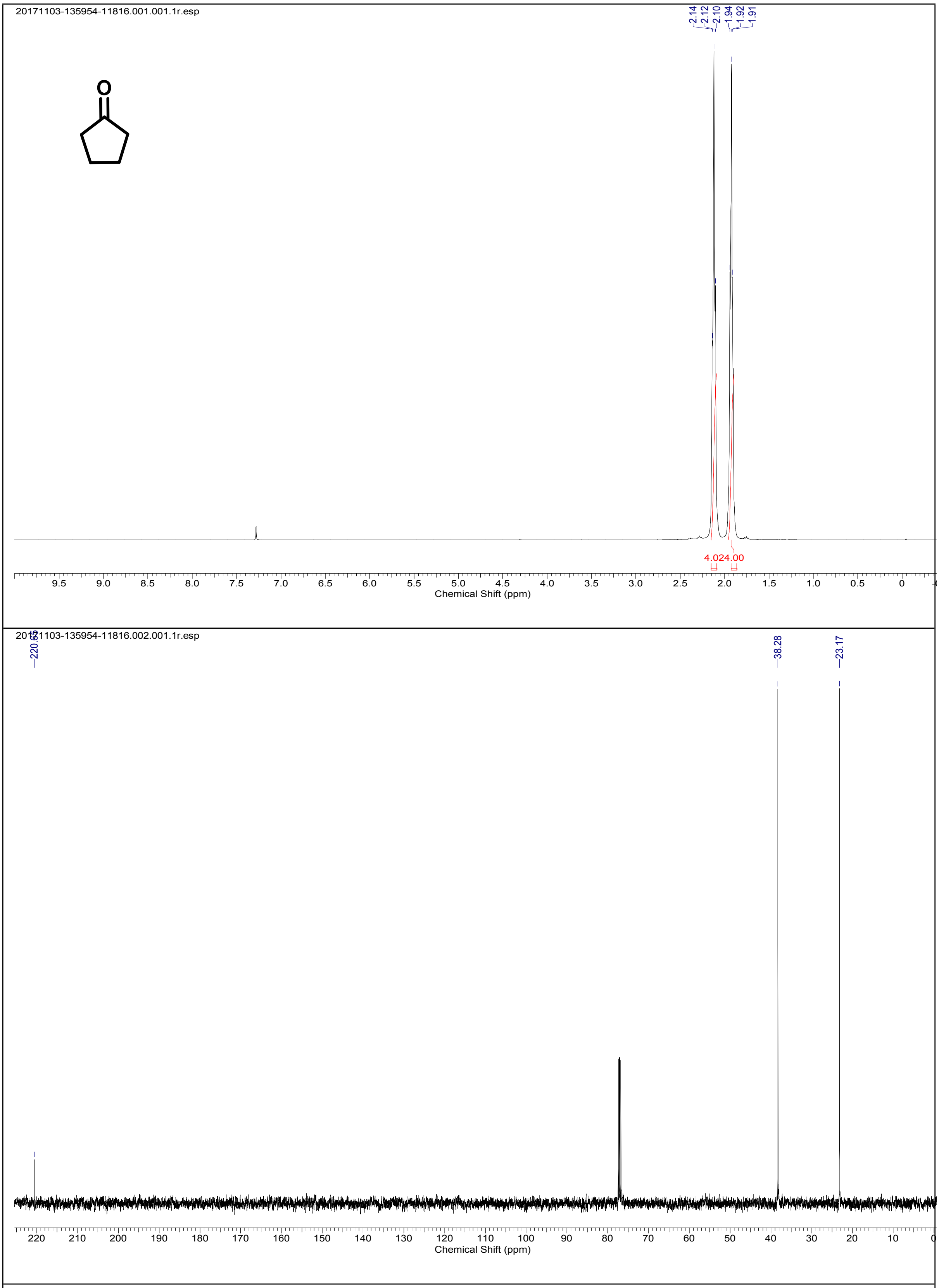

^{1}H and ^{13}C Spectra of 2t

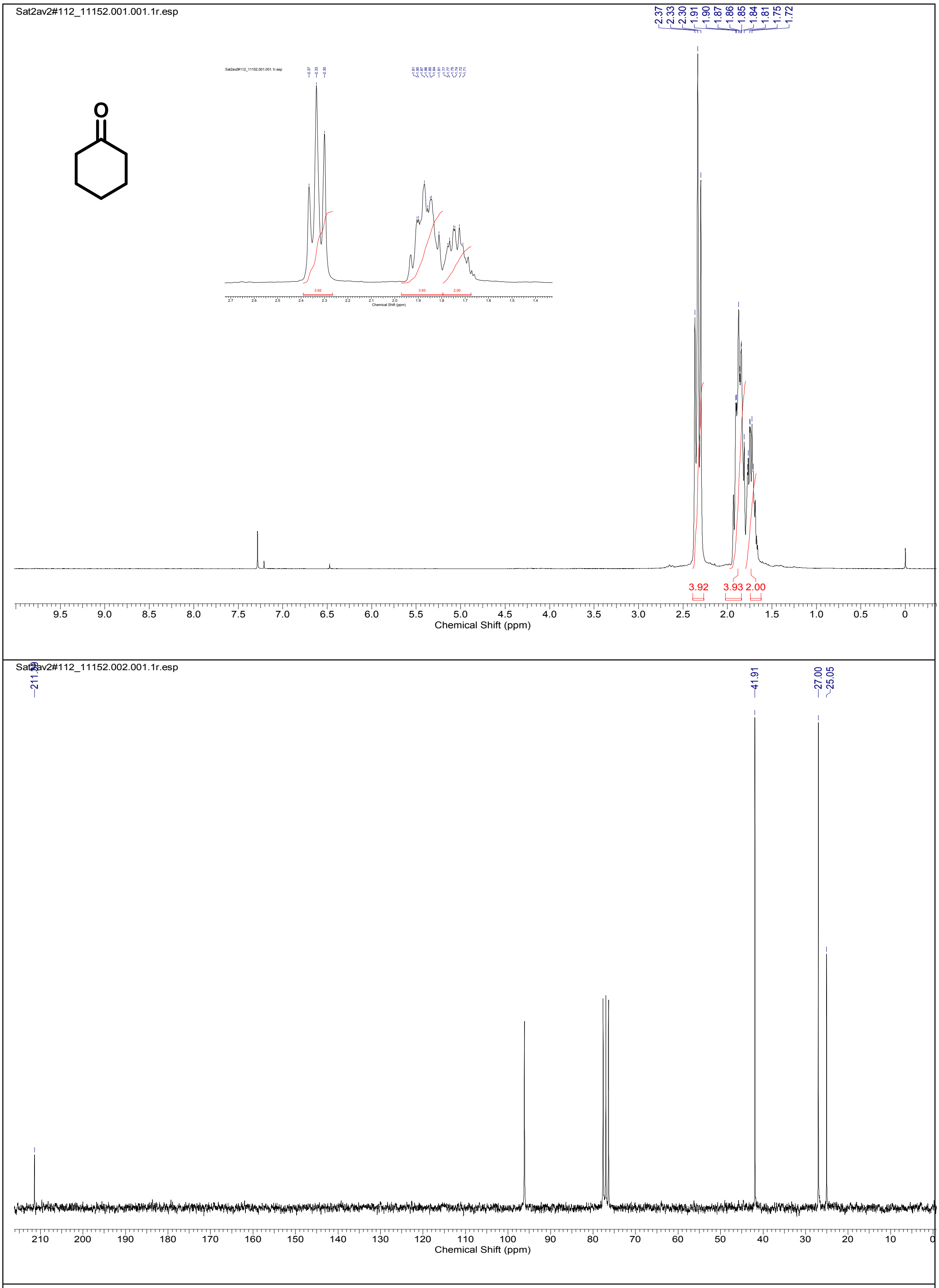

^{1}H and ^{13}C Spectra of 2u

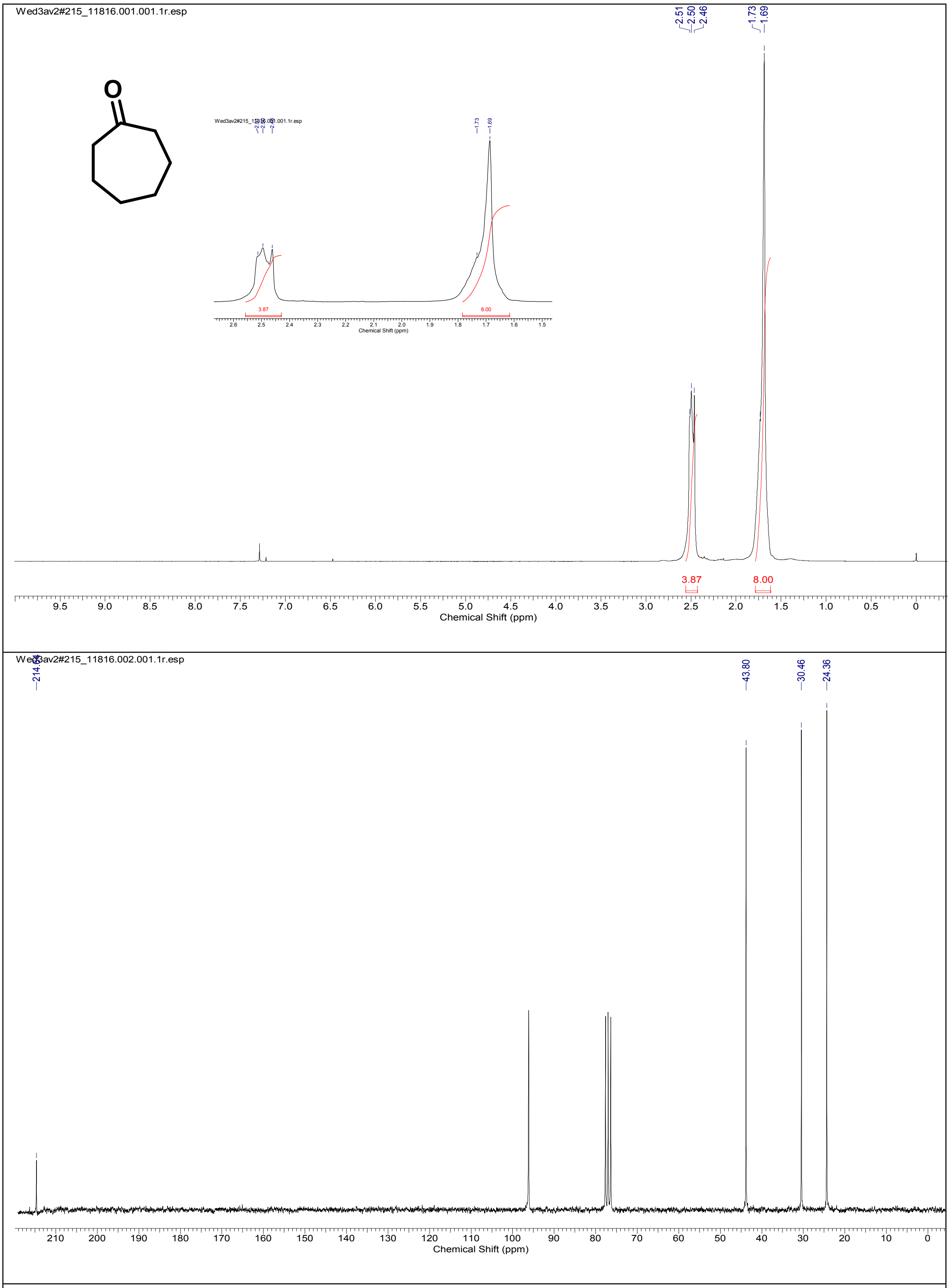

^{1}H and ^{13}C Spectra of 2v

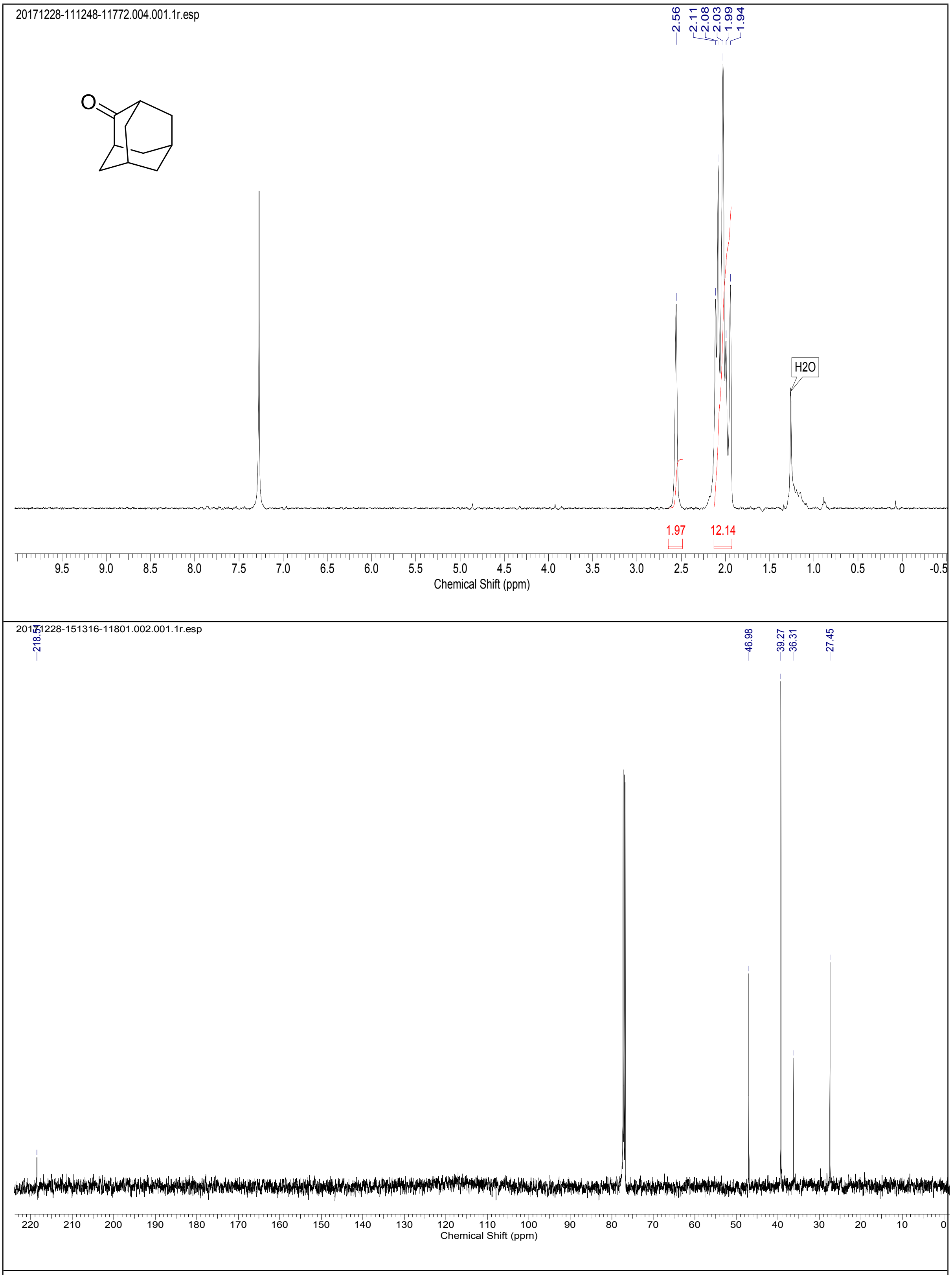

^{1}H and ^{13}C Spectra of 2w

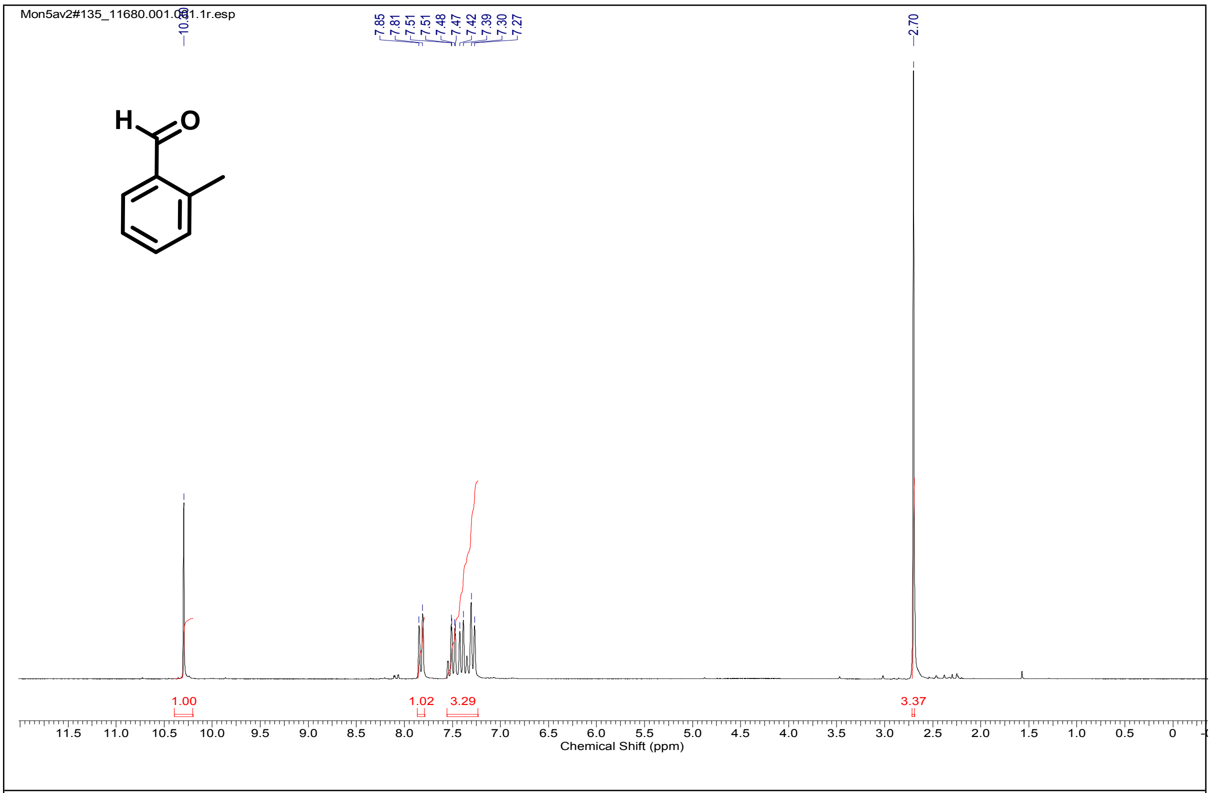

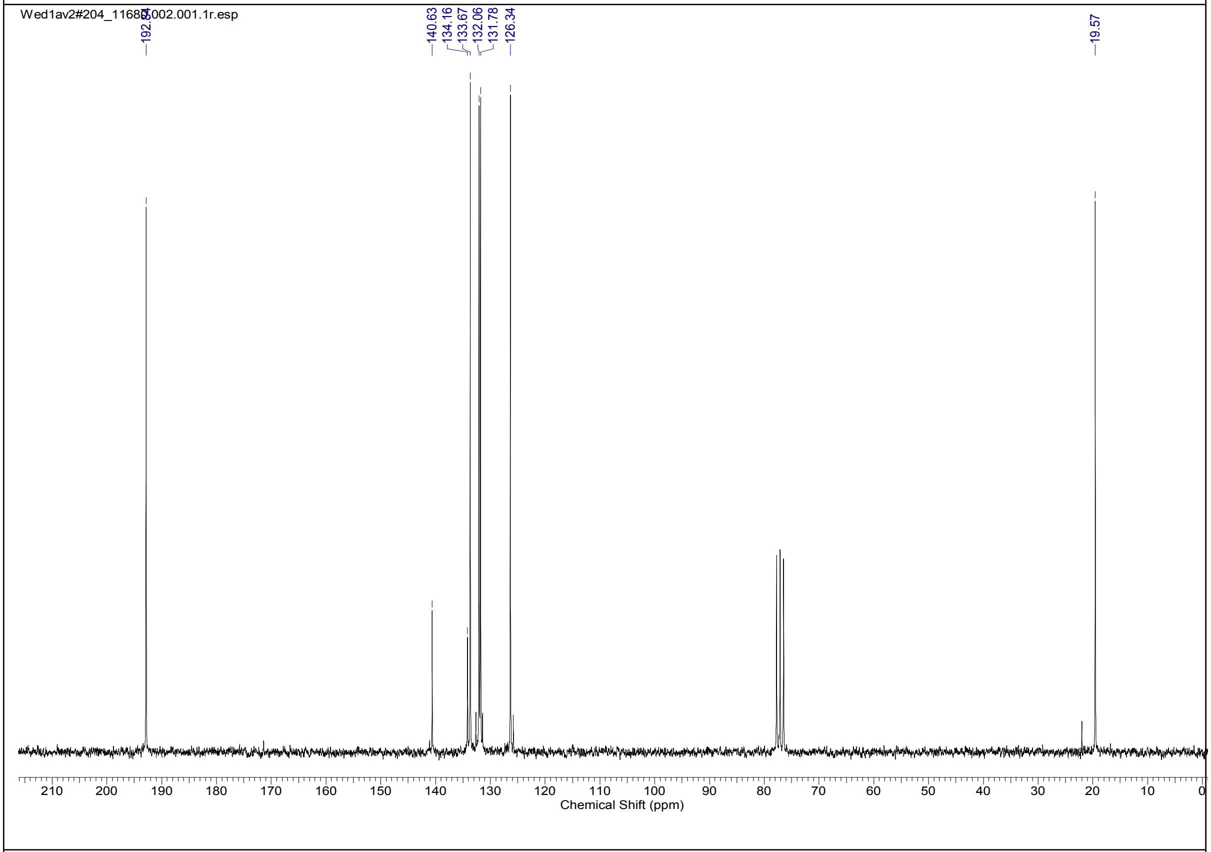

^{1}H and ^{13}C Spectra of 2x

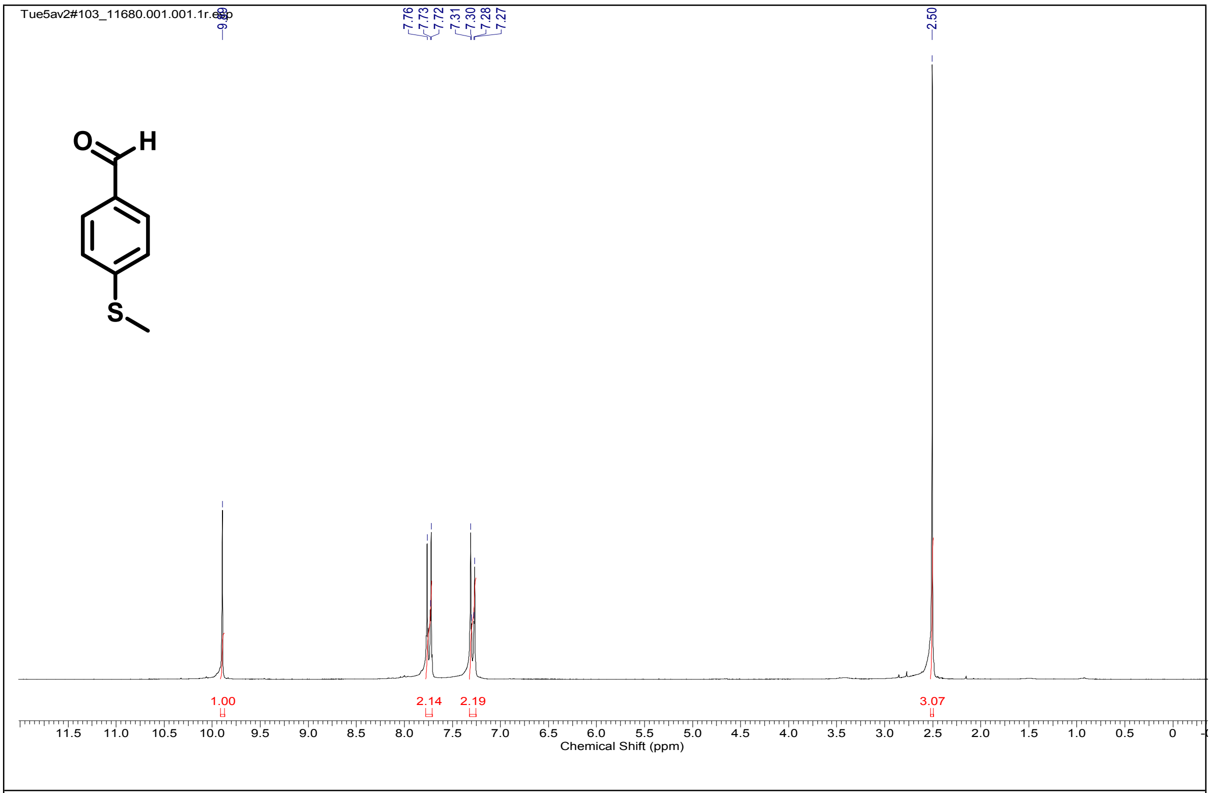

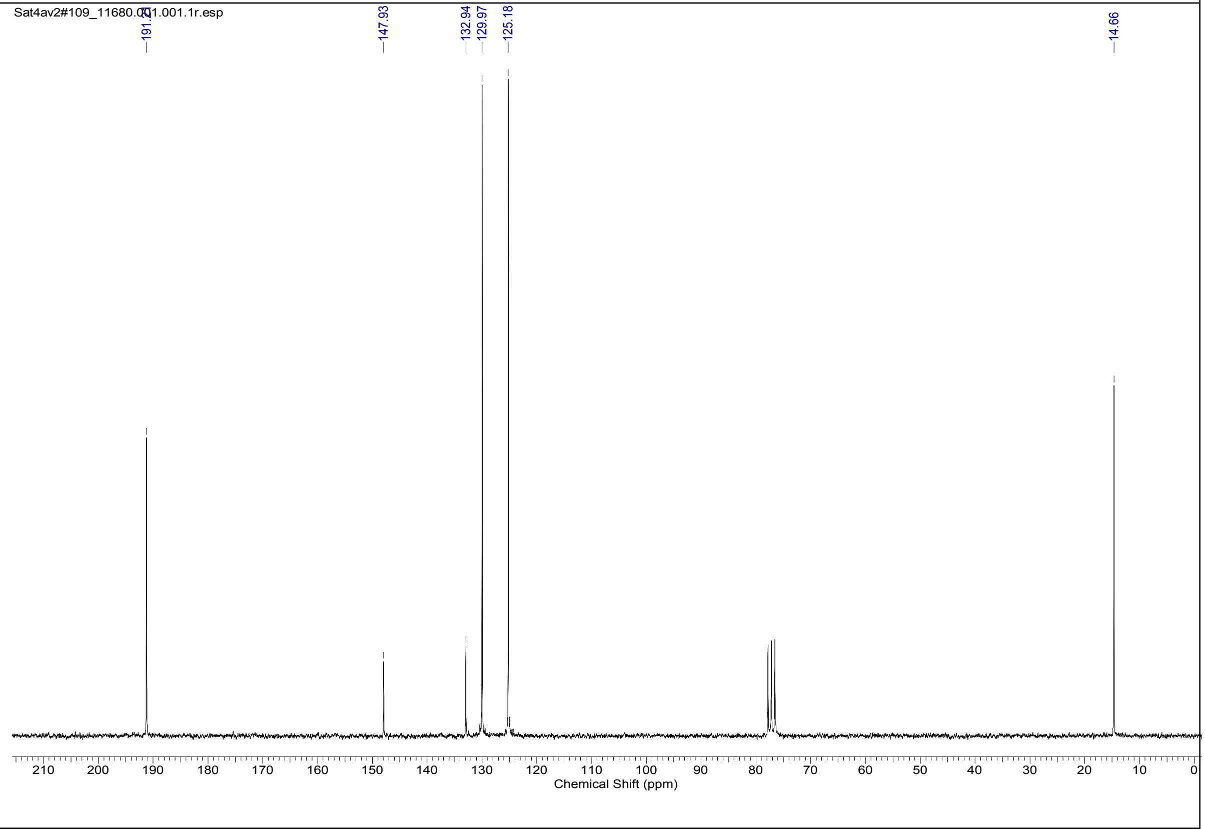

^{1}H and ^{13}C Spectra of 2y

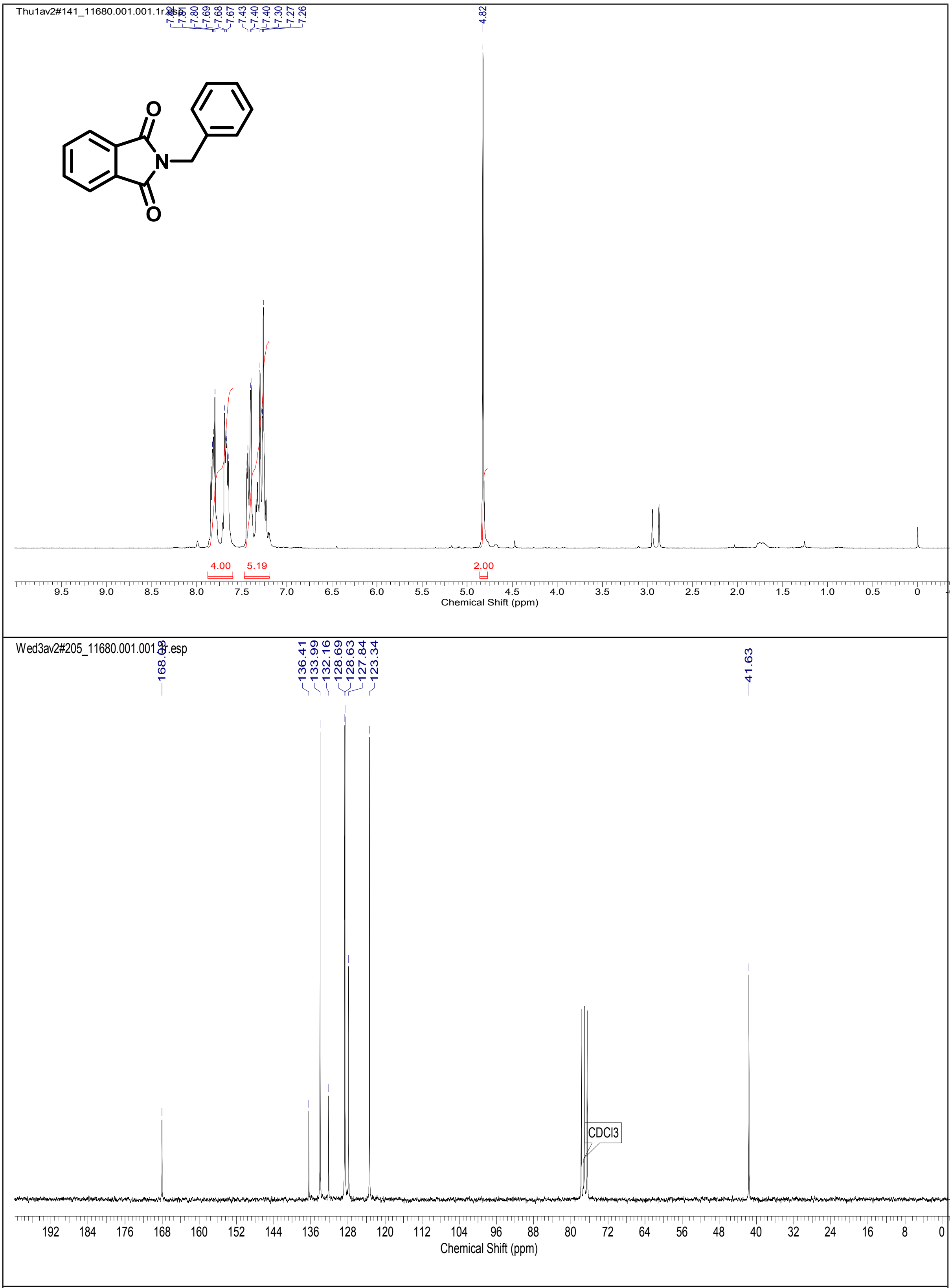

^{1}H and [13]C Spectra of 2-Benzylisoindoline-1,3-dione(p)

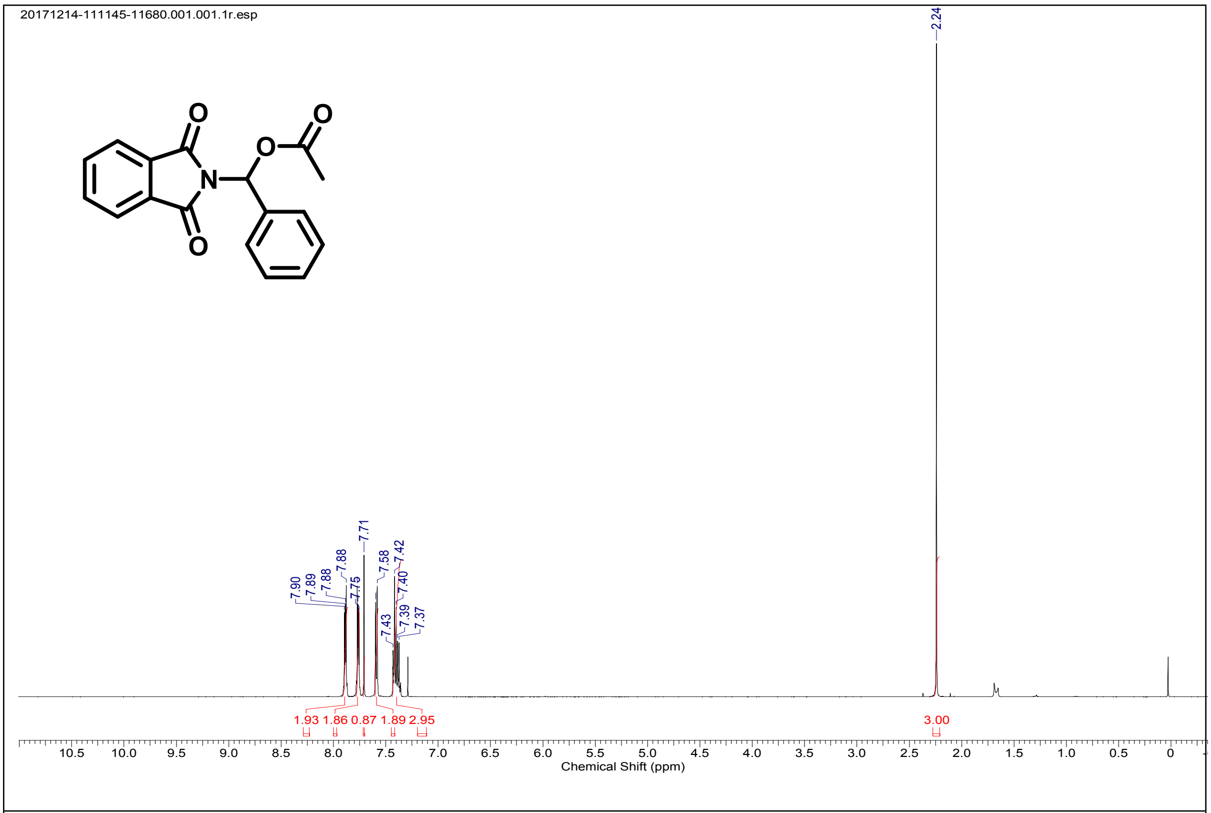

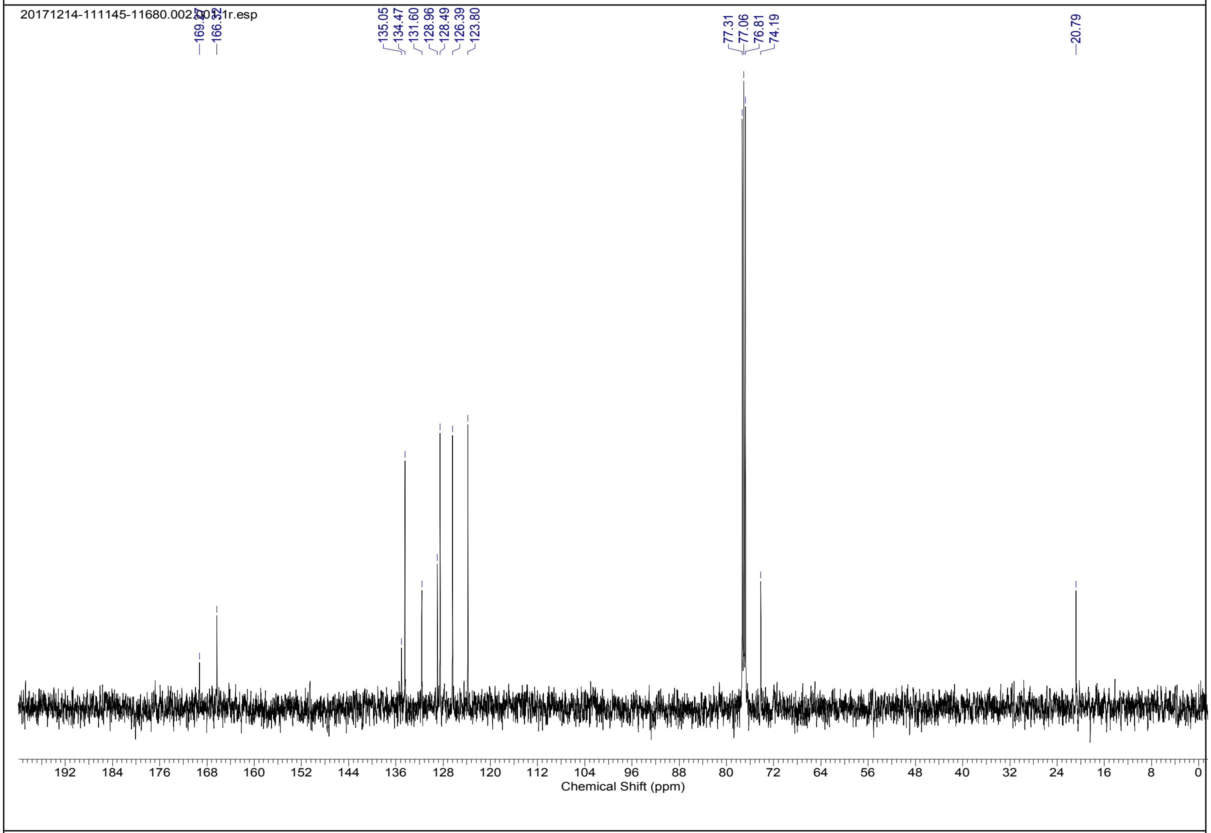

^{1}H and [13]C Spectra of (1,3-dioxoioindoin-2yl) (phenyl)methyl acetate (q)

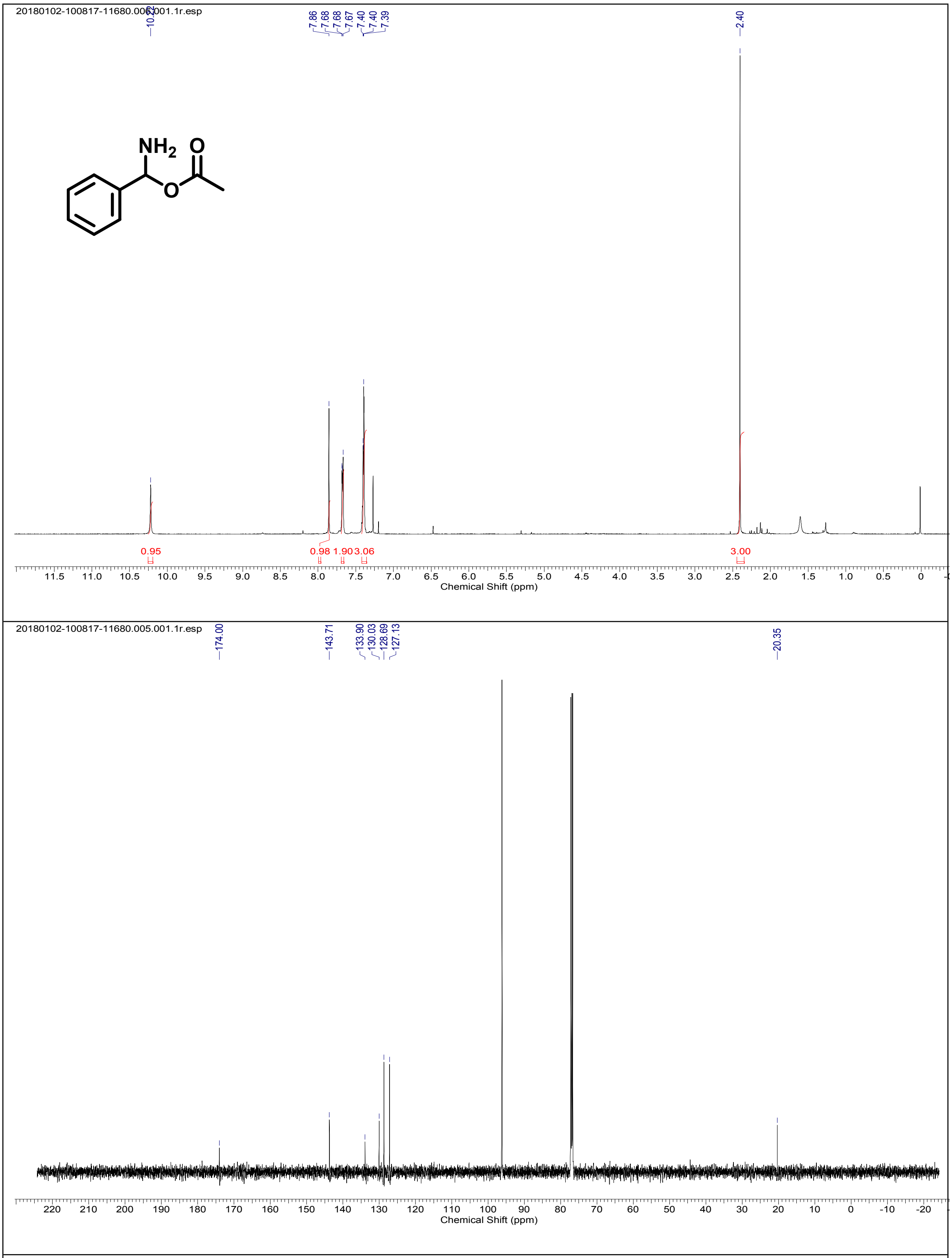

^{1}H and ^{13}C Spectra of Amino(phenyl)methyl acetate (r)
